# Brain health concerns in former rugby players: clinical and cognitive phenotypes

**DOI:** 10.1093/brain/awae416

**Published:** 2025-07-03

**Authors:** Thomas D Parker, Jessica A Hain, Erin J Rooney, Karl A Zimmerman, Ying Lee, Martina Del Giovane, Neil S N Graham, Maneesh Patel, Adam Hampshire, Mathew G Wilson, Daniel Friedland, David J Sharp, Richard J Sylvester

**Affiliations:** Department of Brain Sciences, Division of Medicine, Imperial College London, London W12 0BZ, UK; UK Dementia Research Institute Centre for Care Research & Technology, Imperial College London and University of Surrey, London W12 0BZ, UK; Department of Neurodegenerative Disease, The Dementia Research Centre, UCL Queen Square Institute of Neurology, London WC1N 3BG, UK; Department of Brain Sciences, Division of Medicine, Imperial College London, London W12 0BZ, UK; UK Dementia Research Institute Centre for Care Research & Technology, Imperial College London and University of Surrey, London W12 0BZ, UK; Department of Brain Sciences, Division of Medicine, Imperial College London, London W12 0BZ, UK; UK Dementia Research Institute Centre for Care Research & Technology, Imperial College London and University of Surrey, London W12 0BZ, UK; Institute of Sport, Exercise and Health (ISEH), University College London W1T 7HA, UK; Department of Brain Sciences, Division of Medicine, Imperial College London, London W12 0BZ, UK; UK Dementia Research Institute Centre for Care Research & Technology, Imperial College London and University of Surrey, London W12 0BZ, UK; Department of Brain Sciences, Division of Medicine, Imperial College London, London W12 0BZ, UK; UK Dementia Research Institute Centre for Care Research & Technology, Imperial College London and University of Surrey, London W12 0BZ, UK; Institute of Sport, Exercise and Health (ISEH), University College London W1T 7HA, UK; Department of Brain Sciences, Division of Medicine, Imperial College London, London W12 0BZ, UK; UK Dementia Research Institute Centre for Care Research & Technology, Imperial College London and University of Surrey, London W12 0BZ, UK; Department of Brain Sciences, Division of Medicine, Imperial College London, London W12 0BZ, UK; UK Dementia Research Institute Centre for Care Research & Technology, Imperial College London and University of Surrey, London W12 0BZ, UK; Centre for Injury Studies, Imperial College London, London W12 0BZ, UK; Department of Brain Sciences, Division of Medicine, Imperial College London, London W12 0BZ, UK; Department of Brain Sciences, Division of Medicine, Imperial College London, London W12 0BZ, UK; Department of Neuroimaging, Kings College London, London SE5 8AF, UK; Institute of Sport, Exercise and Health (ISEH), University College London W1T 7HA, UK; HCA Healthcare Research Institute, London W1T 7HA, UK; Department of Brain Sciences, Division of Medicine, Imperial College London, London W12 0BZ, UK; Institute of Sport, Exercise and Health (ISEH), University College London W1T 7HA, UK; Department of Brain Sciences, Division of Medicine, Imperial College London, London W12 0BZ, UK; UK Dementia Research Institute Centre for Care Research & Technology, Imperial College London and University of Surrey, London W12 0BZ, UK; Centre for Injury Studies, Imperial College London, London W12 0BZ, UK; Institute of Sport, Exercise and Health (ISEH), University College London W1T 7HA, UK; Acute Stroke and Brain Injury Unit, National Hospital for Neurology and Neurosurgery, Queen Square, London WC1N 3BG, UK

**Keywords:** rugby, TBI, TES, concussion, behaviour, cognition

## Abstract

Epidemiological studies have shown that elite rugby players are at greater risk of neurodegenerative disease in later life, with post-mortem studies conducted in ex-players demonstrating the presence of neuropathologies related to repetitive head impacts, such as chronic traumatic encephalopathy. However, detailed prospective data establishing the clinical presentation of former rugby players with brain health concerns are lacking. In particular, the rates of traumatic encephalopathy syndrome, the clinical correlate of chronic traumatic encephalopathy, and the relationship between clinical outcomes and repetitive head impacts are unknown.

Two hundred former elite rugby players with brain health concerns and 33 matched healthy control subjects were assessed. Self-reported concussion history, career duration, player position, self-rated scales of depression, anxiety, sleep quality, post-concussion symptoms and quality of life, self and informant ratings of neuropsychiatric symptoms and executive function behaviours, were obtained. Formal cognitive testing, traumatic encephalopathy syndrome classification and 3 T MRI were performed.

Former players had a median age of 44 years (90.5% male, median career length = 10.5 years, median self-reported career concussions = 7); 63% were forwards and 37% were backs. Ex-players had elevated scores compared to controls on all symptom scales except sleep quality. Despite frequent subjective memory complaints, performance on cognitive testing did not significantly differ from controls. No players fulfilled criteria for dementia. Twenty-four former players fulfilled research criteria for traumatic encephalopathy syndrome (seven with cognitive impairment, 12 with neurobehavioral dysregulation, five with both). Provisional levels of certainty for chronic traumatic encephalopathy were relatively low (21 ‘suggestive’, three ‘possible’, zero ‘probable/definite’). Forwards and those with higher self-reported concussions were more likely to be classified as having traumatic encephalopathy syndrome based on neurobehavioral disturbance. Symptom burden (depressive and anxiety symptoms, post-concussion symptoms, behaviour rating of executive dysfunction, and neuropsychiatric symptom severity) was higher in those with higher self-reported concussions but was unrelated to years of play or position played. Cavum septum pellucidum was visible on structural imaging in 24% of players (12% in controls) and was more common in the high compared to low concussion group (32% versus 16%).

In summary, former elite rugby players in mid-life had significant symptom burden, especially those self-reporting more concussions. In contrast, objective cognitive impairments and traumatic encephalopathy syndrome were relatively uncommon and there was no evidence of dementia. These results provide insights into the clinical presentations of former elite rugby players with brain health concerns during mid-life and highlight the complex relationship between symptoms, cognition and head impact exposure.

## Teaser

Parker *et al.* studied a group of 200 former elite rugby players with brain health concerns in mid-life and found that whilst symptoms related to anxiety, depression and memory were common, performance on objective memory and cognitive tests was similar to that of healthy individuals matched for age.

## Introduction

Rugby is a collision sport associated with repetitive head impacts (RHI), increased rates of traumatic brain injury (TBI), and symptoms of concussion.^1,2^ Data from large electronic health record databases suggest former rugby players are at a greater risk of dementia and neurodegenerative disease.^3^ In addition, self-reported concussion has been associated with worse cognitive performance in former elite-level male rugby union players over the age of 80.^4^ There is also emerging evidence from current players that active elite rugby participation is associated with a range of biomarker alterations indicative of adverse effects on brain health.^2,5,6^ However, to date, large scale research clinically characterizing former players with brain health concerns at mid-life is lacking. This research is required to understand the range of clinical issues experienced in this population, their relationship to head impact exposure and other risk factors for neurodegeneration and whether targeted interventions in this group are potentially effective.

TBI can initiate a range of neurodegenerative pathologies and there has been particular interest in the development of chronic traumatic encephalopathy (CTE), a neuropathological entity characterized by a distinctive pattern of perivascular accumulation of phosphorylated tau species in cortical grey matter sulci, primarily in frontal and temporal regions that is typically only observed in individuals with a history of RHI.^7,8^ Research has largely focused on the risk of CTE in American football, with career duration and accelerometer-based estimates of cumulative RHI exposure correlating with CTE pathology. Acute symptoms associated with individual head impacts, commonly referred to as ‘concussions’ (that don't necessarily indicate TBI) have not been shown to predict later identification of CTE pathology.^9-12^ Although relatively limited in number, there are also reports of CTE pathology being present in former rugby players at post-mortem, including in players as young as 23 years old, with CTE pathology more common in those with longer career durations.^13-19^

Currently, there are no specific biomarkers of CTE pathology to assist with diagnosis. Research diagnostic criteria have been developed based on clinical phenotypes of individuals with a history of repetitive head impacts that aim to predict the likelihood of CTE pathology. This clinical correlate of CTE, termed traumatic encephalopathy syndrome (TES), is currently proposed to have two core clinical features: progressive neurobehavioral dysregulation and progressive cognitive impairment, but also additionally incorporates delayed onset, motor signs and psychiatric features as supportive features.^20^ Although not without its limitations, this framework represents an advance in our ability to clinically characterize individuals with repetitive impact exposure and concerns regarding their brain health.

To date, there has been no large-scale research detailing the demographic, behavioural, cognitive and psychiatric profiles of former rugby players and the relationships between these measures and head impact exposure. In addition, there are currently no data estimating the prevalence of TES in cohorts of former rugby players. Here we report cross-sectional baseline analysis of 200 elite former UK-based rugby players aged 30–61 years old who have undergone detailed clinical and cognitive assessment in a brain health clinic setting with the following aims: (i) to describe the demographic profile of this cohort of former rugby players; (ii) to investigate the extent of clinical symptoms relevant to brain health within this cohort; (iii) to characterize the cognitive profiles and the prevalence of objective cognitive impairment in this cohort; (iv) to estimate the prevalence of TES and dementia within this cohort of former rugby players; and (v) to examine the relationship between proxies of head injury exposure and clinical outcomes.

## Materials and methods

### Study design

Participants were recruited from a specialist brain health clinic for former players with concerns regarding their brain health based at the Institute of Sport, Exercise and Health, London, UK. The clinic was advertised via a variety of channels. Former players were entered into the clinic via self-referral. The clinic includes prospective collection of data via a structured clinical, cognitive and biomarker assessment. The full protocol has been described in detail previously.^21^ At the initial clinic visit, all players were asked for their consent for their data to be used for research purposes. Consent to research was optional and individuals who did not consent were still able to undergo assessment in the clinic. The rugby component of the study is funded by the Rugby Football Union (RFU) and Premiership Rugby, but all research and analysis are conducted independently from the study funders. Ethical approvals have been granted by the Camberwell St Giles Research Ethics Committee (REC no: 17/l0/2066). All participants provided written informed consent.

### Participants

Two hundred former elite level rugby players (age range 30 to 61) were assessed. Former players must have participated in rugby to an elite level, which was defined as having at least one rugby union or rugby sevens international appearance, and/or being contracted for at least one full season in the highest-level male and female domestic leagues in England (Premiership Rugby and Premiership Women's Rugby, respectively), and/or the second highest male domestic league in England (RFU Championship). Exclusion criteria included an inability or unwillingness to participate in the research study or a contraindication to MRI. Thirty-three healthy participants without an elite sports background were also recruited as a control group. None of the control participants reported a history of TBI or major neurological/psychiatric disorders.

### Clinical assessment and head injury exposure

Each participant underwent a semi-structured clinical interview conducted to collect information on playing career, current symptoms and relevant background medical history. A detailed history of head trauma using the BRAIN-Q and the Ohio State University TBI Identification Method was also performed.^22,23^ All participants underwent blood pressure, height and weight measurement. Bioelectrical impedance analysis derived estimates of body fat percentage and muscle mass were obtained using a TANITA MC-980 body composition scale.

### Participant self-completion questionnaires

Participants were provided with self-completion questionnaires to complete, which collected data regarding: alcohol misuse [Alcohol Use Disorders ID Test (AUDIT)]^24^; drug misuse [Drug Use Disorders ID Test (DUDIT)]^25^; symptoms of depression [Becks Depression Inventory (BDI)]^26^; symptoms of anxiety [Generalised Anxiety Disorder 7 (GAD-7)]^27^; sleep quality [Insomnia Severity Index (ISI) and Pittsburgh Sleep Quality Index (PSQI)]^28,29^; post-concussion symptoms [modified Rivermead post-concussion (mRPO)],^30^ which includes an ‘atypical’ symptom subscale; quality of life [EuroQol 5 Dimensions 5-level (EQ-5D-5L)]^31^; and executive function and behaviour [Behavioural Rating Inventory of Executive Function Adult Version (BRIEF-A)].^32^

### Informant questionnaires

Participants were asked to identify a person who knows them well (e.g. partner/relative/close friend) to complete informant ratings of executive function and behaviour using the BRIEF-A and neuropsychiatric symptoms using the Neuropsychiatric Inventory (NPI).^33^

### Cognitive testing

A standardized cognitive testing battery was administered to each participant by a trained researcher. The test battery included: an estimate of premorbid functioning (FSIQ)^34^; performance validity testing (dot counting score and reliable digit span)^34,35^; measures of executive function [Trail making test, B versus A ratio and Delis-Kaplan Executive Function System (D-KEFS) inhibition switching versus baseline contrast]^36,37^; an auditory memory composite score [Repeatable Battery for the Assessment of Neuropsychological Status (RBANS) and Wechsler Memory Scale, Fourth Edition (WMS IV)]^38,39^; a processing speed index score (WAIS IV)^34^; a working memory index score (WAIS IV)^34^; an attention index score (RBANS)^39^; a language index score (RBANS)^39^; and a visuospatial index score (RBANS).^39^

Participants also completed a complementary battery of five computerised tasks from the Cognitron platform on the same electronic touchscreen tablet.^40-42^ This battery consisted of tests of immediate and delayed memory (Objects immediate memory and Objects delayed memory); an adapted version of the Tower of London test^43^; a 2D mental manipulation task (2D Manipulations); and processing speed tasks (Simple reaction time and Choice reaction time). The Objects memory, Tower of London and 2D Manipulation tasks resulted in a primary accuracy-based score as well as secondary response time score.

### Clinical MRI sequences

Participants underwent MRI brain scanning that was clinically reported by an experienced consultant neuroradiologist (M.P.). Clinical sequences included volumetric T_1_, FLAIR imaging, as well as blood sensitive susceptibility weighted imaging (SWI) sequences to detect microhaemorrhages. All T_1_ images were reviewed independently in a blinded manner (T.D.P.) to assess presence or absence of a cavum septum pellucidum (CSP), as this is linked to repeated head injury exposure in other contexts.^44^ A definite CSP was defined as grade 2 or above (i.e. clear evidence of CSF signal between the separated leaves of the septum pellucidum).^44^

### Traumatic encephalopathy syndrome classification

Each former player assessed underwent TES classification using the National Institute of Neurological Disorders and Stroke Consensus Diagnostic Criteria for Traumatic Encephalopathy Syndrome framework.^20^ This was performed in a multi-disciplinary setting led by consultant neurologists with expertise in cognitive disorders and TBI (D.J.S. and R.S.) and a consultant clinical neuropsychologist (D.F.). The precise criteria are detailed elsewhere, but in brief, to be defined as TES positive the following overarching criteria must be reached in a stepwise fashion: (i) substantial exposure to repetitive head impacts; (ii) cognitive impairment AND/OR neurobehavioural dysregulation AND a progressive course; and (iii) not fully accounted for by other disorders.^20^ To be defined as having cognitive impairment there must be either a self, informant or clinician report of cognitive issues that represents a significant decline from baseline functioning with deficits in episodic memory and/or executive functioning that can be substantiated with objective deficits on formal cognitive testing [at least 1.5 standard deviations (SD) below appropriate norms accounting for pre-morbid function].^20^ To be defined as having neurobehavioural dysregulation there must be either a self, informant or clinician report of behavioural change that represents a significant decline from baseline functioning with symptoms including explosiveness, impulsivity, rage, violent outbursts, and emotional lability.^20^ Given the cross-sectional nature of the dataset, evidence of progressive course was based on the history available by reviewing the clinical notes in detail.

In addition to the core features detailed above, supportive features including a delayed symptom onset from head impact exposure, presence of motor signs, presence of psychiatric features, as well as severity of functional dependence (ranging from fully independent, subtle/mild functional limitation, to mild, moderate and severe dementia) are used to determine a provisional level of certainty for CTE pathology.^20^ Provisional levels of certainty for CTE pathology include: definite CTE; probable CTE; possible CTE; or suggestive of CTE. Definite CTE requires neuropathological confirmation at post-mortem. Probable CTE requires exposure to repetitive head impacts ≥11 years AND objective cognitive impairment AND a minimum of 3 out of 5 of the following criteria: delayed onset; motor signs; one or more psychiatric features; neurobehavioural dysregulation; or a functional dependence level of mild dementia or worse. Possible CTE requires exposure to repetitive head impacts ≥5 years AND objective cognitive impairment AND a minimum of 2 out of 5 of the following criteria: delayed onset; motor signs; one or more psychiatric features; neurobehavioural dysregulation or a functional dependence level of subtle/mild functional limitation or worse. The term, suggestive of CTE, is applied when an individual meets the core clinical TES criteria (i.e. cognitive impairment AND/OR neurobehavioural dysregulation not fully accounted for by other disorders with a progressive course in the context of substantial exposure to repetitive head impacts), but does not fulfil the criteria for possible, probable or definite CTE.^20^

### Estimates of exposure

Former players were binarized into low or high concussion groups based on a median split, with the low concussion group defined by participants who self-reported seven or less concussions during their rugby playing career and the high concussion group defined as participants who self-reported eight or more concussions during their career. A definition of concussion as per the BRAIN-Q questionnaire was provided to each participant as follows:

‘an alteration in brain function, caused by an external force. Symptoms include: a decreased level / loss of consciousness; memory loss (before or after the injury); weakness / temporary paralysis; loss of balance; change in vision (e.g. blurriness, double vision); co-ordination difficulties; numbness; decreased sense of smell; difficulty understanding what others are saying; difficulty communicating with others; confusion, disorientation, or slowed thinking. Loss of consciousness is not required for a concussion to be diagnosed.’

Career duration is a key part of the TES criteria and has been shown as a predictor of CTE pathology in rugby players and athletes exposed to repetitive head impacts more generally.^19,20^ Career duration (number of years), was calculated by subtracting first year of elite play from year of retirement. In addition, former players were binarized into forwards or backs depending on their primary position. There is evidence that forwards and backs exhibit different anthropometric and physiological characteristics, which is likely a reflection of differences in specific demands during training and matches.^45^ Forwards are involved in more tackles, tackle assists, breakdown entries and contact events.^46^ In addition, there is evidence from instrumented mouth guards, that forwards experience more in-game head impacts than backs.^47,48^

### Missing data

Two former players were unable to provide an estimate of self-reported concussions. One former player was unable to complete the MRI scan due to claustrophobia. One former player's neuropsychology testing results were excluded from analyses as they reported being under the influence of alcohol during testing. One former player was missing D-KEFS colour naming time, another was missing WMS-IV verbal paired associates due to anxiety over performance. Twelve of the former players were unable to complete the computerized cognitive testing battery due to time limitations on the day. Body composition measurement was not performed for 23 players as the decision to acquire these measures were instituted after the study has started. The following number of self-completion/informant questionnaires were not returned by the former players: AUDIT *n* = 8, DUDIT *n* = 8, EuroQol 5dim *n* = 8, BDI *n* = 7, GAD7 *n* = 7, modified Rivermead *n* = 9, PSQI *n* = 7, ISI *n* = 8, BRIEF-A *n* = 9, informant-report BRIEF-A *n* = 22, and NPI-Q *n* = 33. The following number of self-completion/informant questionnaires were not returned by the control group: DUDIT *n* = 3, BDI *n* = 1, modified Rivermead *n* = 1, ISI *n* = 1, self-report BRIEF-A *n* = 1, informant-report BRIEF-A *n* = 2, NPI-Q *n* = 3. A complete case approach was used throughout the statistical analysis.

### Statistical analysis

To assess the demographic profile of the cohort we examined: age; sex; years of education; ethnicity; weight; height; body mass index; body composition; smoking; blood pressure; current exercise levels; alcohol use; illicit drug use; career length; player position; and player position. Where data were available univariate analyses looking at differences between former players and controls, using Wilcoxon rank sum test, Spearman's rank correlations, Chi-squared tests, logistic regression, T-test and Pearson's correlation as appropriate.

To assess symptom burden, raw scores from self and informant completed symptom questionnaires were compared between ex-players and controls using Wilcoxon rank sum tests. In addition, the proportion of former players and controls scoring above cut-offs felt to be statistically significant was compared using Chi-squared tests. Similar analyses were performed within the rugby players only, investigating the relationship between symptom burden and estimates of exposure (self-reported concussion, career length and player position) using Wilcoxon rank sum test, Spearman's rank correlations and Chi-squared tests as appropriate.

To assess objective cognitive performance, scores from each standardized cognitive test were compared between former players and controls using linear regression analyses with adjustment for age, sex and years of education. In addition, logistic regression models adjusted for age and sex comparing the proportion of former players and controls performing at a level felt to indicate significant impairment on that test (e.g. ≥1.5 SD below each participant's FSIQ) were also performed. Similar analyses were performed within the rugby players only, investigating the relationship between cognitive performance and estimates of exposure (self-reported concussion, career length and player position).

Performance on the computerized Cognitron tasks was first estimated using the raw accuracy and reaction time scores. Group comparisons between former players and controls were performed using linear regression analyses with adjustment for age, sex and years of education, or generalized linear model with gamma log link function in metrics with significantly positively skewed distributions. Similar analyses were also performed within the former rugby players using the three predictor variables hypothesized to relate to head impact exposure (self-reported concussion, career length and player position). Subsequently, cognitive performance on the computerized tasks was studied converting the raw accuracy and reaction time scores into ‘Deviation from Expected’ (DfE) scores. The latter was done via modelling a large normative dataset (mean *n* = 140, 302) collected via the Great British Intelligence Test, a collaborative project with the British Broadcasting Corporation Horizon programme that started in December 2019.^41,49^ Specifically, linear models were trained to the normative dataset to predict each score based on a range of demographic factors (i.e. age, age squared, gender, education, language, handedness and device). The trained set was then applied to the participants’ dataset to assign each participant the expected score for a cognitively healthy person with the same demographic characteristics. The difference between the participant's expected and observed score, divided by the standard deviation of the population dataset, represents the DfE score. The same investigations described above were conducted on the DfE scores. General linear models were conducted to compare former rugby players with controls, and to investigate the relationship between cognitive performance and the three predictor variables hypothesized to relate to head impact exposure. Demographic variables were not included in these models, as they had already been accounted for during DfE score estimation.

For the primary analyses of interest, i.e. those comparing former rugby players and controls, a standard statistical threshold of *P* < 0.05 was used. For secondary analyses investigating the effect of career duration, player position and self-reported concussion on symptom burden and cognitive performance, false discovery rate (FDR) correction for multiple comparisons were performed to reduce the risk of type I error.

## Results

### Demographics

Participant characteristics are displayed in Table 1. Compared to control subjects, former players were well matched in terms of age and sex but spent less time in full-time education. Former players had higher weight, height, body mass indices and muscle mass compared to controls. There was no difference in body fat percentage. Former players reported more hours of exercise per week, but also had evidence of higher levels of alcohol consumption.

**Table 1:** Participant characteristics in the advanced BRAIN (BiomaRker, advanced imaging and neurocognitive) health study

	Former rugby player (*n* = 200)	Non-sporting control (*n* = 33)	*P*-value	Association with career duration
Age, years, median (IQR)	44.0 (38.8 to 51.0)	47.0 (40.0 to 56.0)	0.14^a^	*Rho* = 0.2, *P* = 0.004*^,b^
Male sex, *n* (%)	181 (90.5)	26 (78.8)	0.093^c^	Median duration, males = 11 years, females = 8 years, *P* = 0.022*^,a^
Years of education, median (IQR)	16.0 (14.0 to 17.0)	17.0 (16.0 to 18.0)	0.003*^,a^	*Rho* = −0.22, *P* = 0.002*^,b^
Ethnicity, *n* (%)			0.69^c^	Median duration, white ethnicity = 10.5 years, other ethnicities = 9.5 years, *P* = 0.4^a^
White	190 (95.0)	32 (97.0)	–	–
Black Caribbean	1 (0.5)	0 (0.0)	–	–
Black African	3 (1.5)	0 (0.0)	–	–
Other ethnic group (including ‘mixed’)	4 (2.0)	0 (0.0)	–	–
Not stated	2 (1.0)	1 (3.0)	–	–
Weight, kg, median (IQR)	101.8 (92.2–113.9)	82.6 (73.2–90.2)	<0.001*^,a^	*Rho* = 0.17, *P* = 0.016*^,b^
Height, cm, mean (SD)	185.4 (9.1)	177.4 (9.8)	<0.001*^,d^	*r* = 0.085, *P* = 0.23^e^
BMI, median (IQR)	29.0 (27.2–32.6)	26.0 (23.2–28.0)	<0.001*^,a^	*Rho* = 0.16, *P* = 0.027*^,b^
Body fat (%), median (IQR)	23^f^ (19–28)	23 (19–29)	0.7^a^	*Rho* = 0.03, *P* = 0.74^b^
Muscle mass (kg), median (IQR)	75^f^ (69–81)	62 (54–66)	<0.001*^,a^	*Rho* = 0.13, *P* = 0.075^b^
Smoking, never, *n* (%)	181 (91%)	26 (79%)	0.068^c^	Median duration, smoked = 11 years, never-smoked 9 years, *P* = 0.2^a^
Hypertension, ≥140/90, *n* (%)	10 (5%)	4^g^ (13%)	0.11^c^	Median duration, hypertension = 11 years, normal BP 10.5 years, *P* = 0.5^a^
Exercise, hours per week, median IQR	4.0^h^ (3.0, 6.5)	2.5 (2.0–4.5)^i^	0.014*^,a^	*Rho* = 0.05, *P* = 0.5^b^
AUDIT raw score, median (IQR)	7^j^ (4–11)	5 (3–8)	0.003*^,a^	*Rho* = 0.03, *P* = 0.65^b^
Alcohol consumption (AUDIT-C), median (IQR)	5^j^ (4–7)	4 (3–5)	0.007*^,a^	*Rho* = 0.09, *P* = 0.22^b^
Alcohol problematic consequences (AUDIT-*P*), median (IQR)	2^k^ (0–4)	0.0 (0–1)	0.005*^,a^	*Rho* = −0.03, *P* = 0.68^b^
Ilicit drug use total score (DUDIT), median (IQR)	0^k^ (0 ± 1)	0 (0 ± 1)^l^	0.6^a^	*Rho* = −0.01*, P* = 0.89^b^
Ilicit drug use (DUDIT ≥1), *n* (%)	24/193 (12.0)	5/30 (15.2)	0.7^c^	Median duration, nil drug use = 11 years, drug use = 8 years, *P* = 0.022*^,a^
Length of professional career, years, median (IQR)	10.5 (8.0–13.0)	–	–	*–*
Forwards (F):backs (B), *n* (%)	126:74 (63%:37%)	–	–	Median duration, forwards = 11 years, backs = 10 years, *P* = 0.23^a^
Previous self-reported concussions in rugby, median (IQR)	7.0^m^ (4.0–20.0)	–	–	*Rho* = 0.06^m^*, P* = 0.37^b^
Microhaemorrhage, *n* (%)	6 (3)^n^	0 (0)	0.67^c^	Median duration, microhaemorrhage present = 12.5 years, absent = 9 years, *P* = 0.3^a^
Cavum septum pellucidum grade 2 or above, *n* (%)	48 (24)^n^	4 (12.1)	0.19^c^	Median duration, CSP present = 11 years, absent = 10 years, *P* = 0.11^a^

*n* = 200 former rugby players and 33 non-sporting control subjects, unless otherwise specified. AUDIT = Alcohol Use Disorder Identification Test; BMI = body mass index; BP = blood pressure; CSP = cavum septum pellucidum; DUDIT = Drug Use Disorder Identification Test; IQR = interquartile range; OR = odds ratio; SD = standard deviation; r = Pearson correlation coefficient; Rho = Spearman’s rank correlation coefficient.

^a^Wilcoxon rank sum test.

^b^Spearman’s rank correlation.

^c^Chi-squared test.

^d^T-test.

^e^Pearson correlation.

^f^ *n* = 177.

^g^ *n* = 32.

^h^ *n* = 197.

^i^ *n* = 31.

^j^ *n* = 192.

^k^ *n* = 193.

^l^ *n* = 30.

^m^ *n* = 198.

^n^ *n* = 199.

^*^ *P*-values < 0.05.

### Player positions, career duration and self-reported concussion

**Figure 1:**
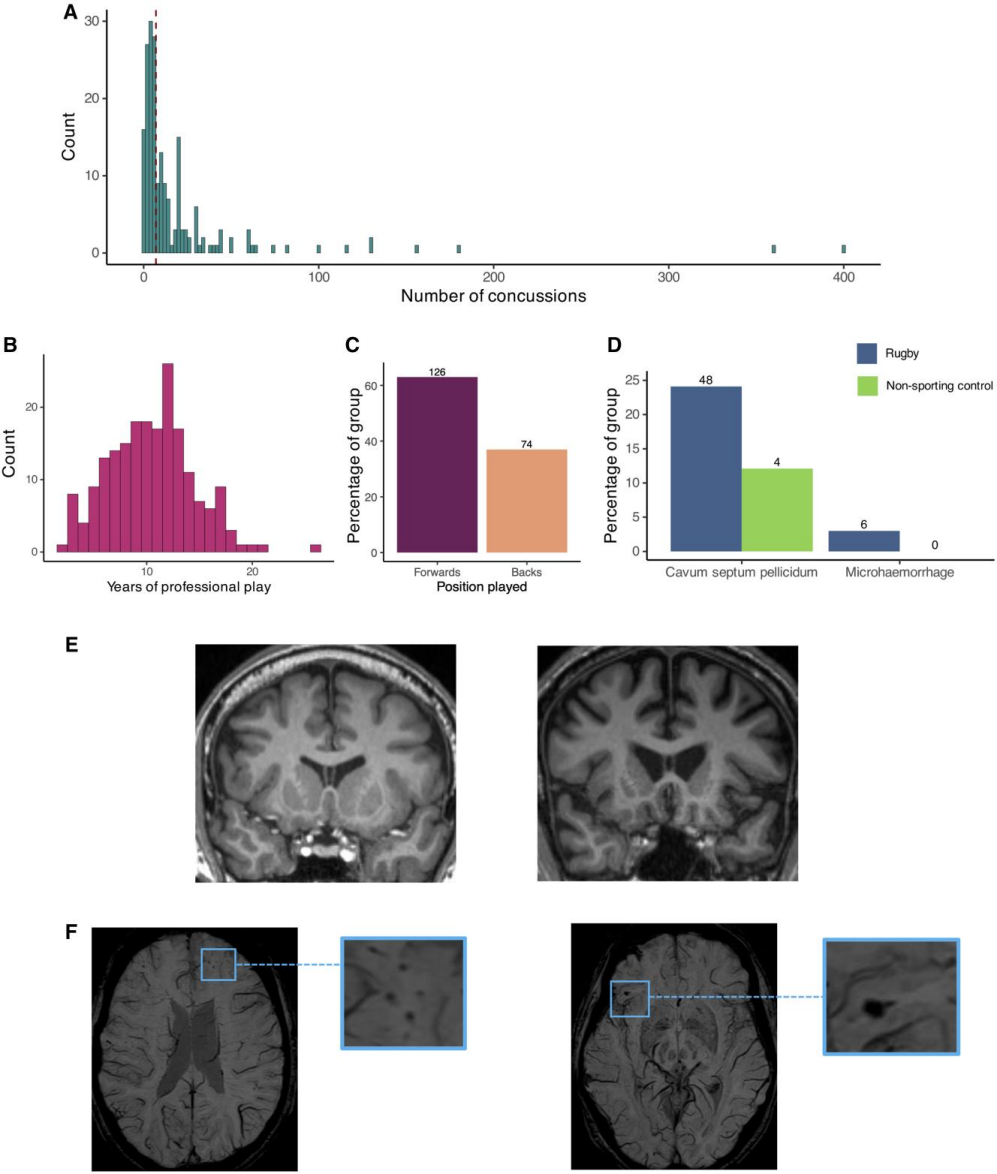
**Rugby career-related statistics and clinical imaging**: (**A**) Histogram showing self-reported concussions within rugby (dashed lined represents median value). (**B**) Histogram showing distribution of career duration. (**C**) Player positions. (**D**) Trauma-related imaging abnormalities. (**E**) Examples of cavum septum pellucidum on T_1_-weighted image. (**F**) Examples of microhaemorrhage on susceptibility weighted imaging (SWI).

The median self-reported concussion count within the rugby group across their career length was seven (IQR = 8 to 20) (Fig. 1B). The median self-reported concussion with loss of consciousness within rugby across their career length was one [interquartile range (IQR) = 0 to 2.3]. Concussions outside of the context of rugby were less frequent, with only 8/200 players self-reporting such instances, with the median number of concussions outside rugby being 0 (IQR = 0 to 1). Median career length was 10.5 years (IQR = 8.0 to 13.0). In terms of player position, 126 of the 200 (63%) former players were forwards, whilst the remaining 74 (37%) were backs (full breakdown in Fig. 1A).

### Clinical imaging findings

Microhaemorrhages were present in 6/199 (3.0%) of the former players on SWI compared to 0/33 (0%) in the control group (χ^2^ = 0.16, df = 1, *P* = 0.82). CSP was seen in 48/199 (24.0%) of the former players (grade 2 or above). The proportion of CSP in controls was lower 4/33 (12.1%), although this was not a statistically significant difference (χ^2^ = 0.17, df = 1, *P* = 0.19). White matter hyperintensities were reported in 20/199 (10.3%) of the ex-players and 4/33 (12.1%) controls (χ^2^ = 0.003, df = 1, *P* = 0.96). None of the controls were reported to have atrophy on their structural imaging, whereas 2/199 (1.0%) former players (χ^2^ = 0.00, df = 1, *P* = 1) were reported to have some greater than expected volume loss. Other incidental abnormalities unrelated to trauma were seen in 3/199 (1.5%) of the ex-players and 1/33 (3.0%) of controls (χ^2^ = 0.00, df = 1, *P* = 1), whilst 134/199 (69.4%) of former players and 27/33 (81.8%) of controls had a radiological report with no abnormalities (χ^2^ = 2.15, df = 1, *P* = 0.14).

### Former players have higher symptom burden than healthy controls

**Figure 2:**
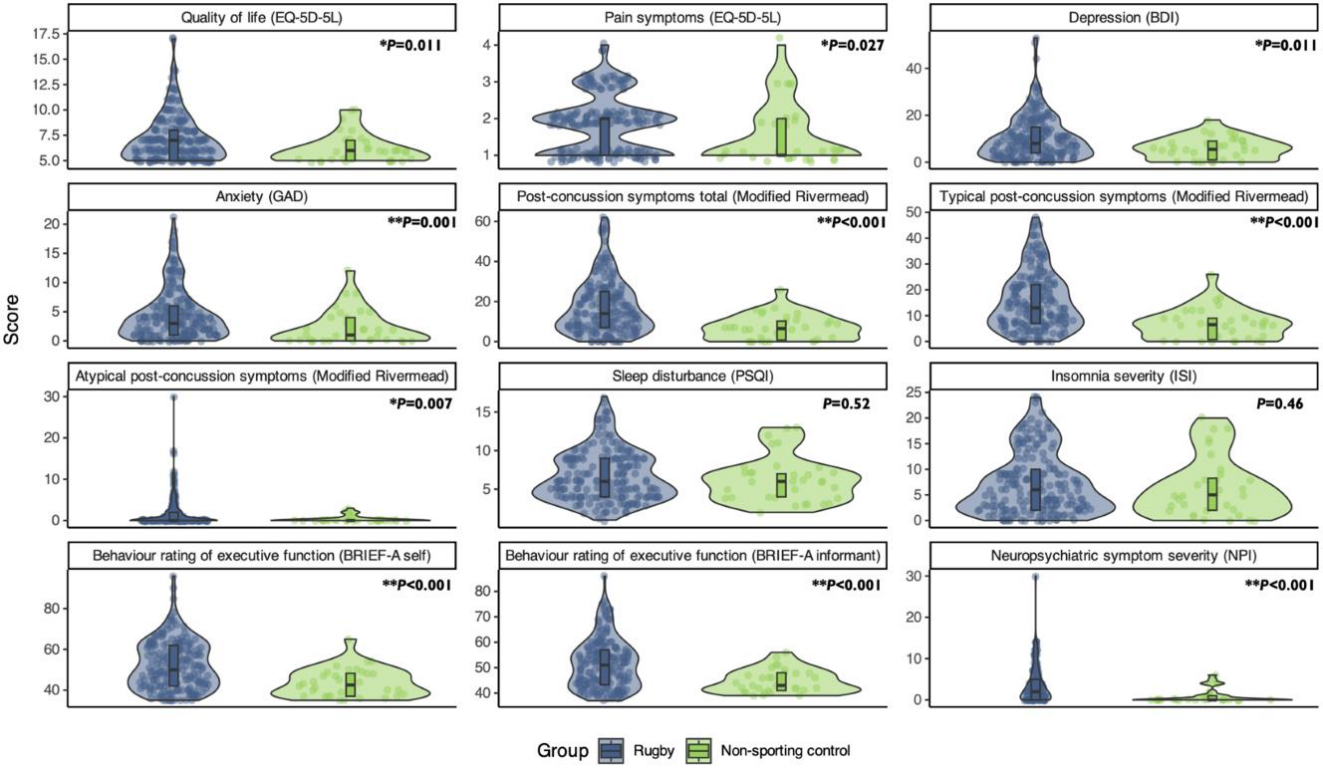
**Violin plots comparing symptom burden in former rugby players and control subjects**. Scales included those assessing symptoms of depression [Becks Depression Inventory (BDI)]^26^; symptoms of anxiety [Generalised Anxiety Disorder 7 (GAD-7)]^27^; sleep quality [Insomnia Severity Index (ISI) and Pittsburgh Sleep Quality Index (PSQI)]^28,29^; post-concussion symptoms [modified-Rivermead Post-concussion (mRPO)],^30^ which includes an ‘atypical’ symptom sub-scale; quality of life (EuroQol 5 Dimensions 5-level, EQ-5D-5L)]^31^; executive function and behaviour [Behavioural Rating Inventory of Executive Function Adult Version (BRIEF-A)].^32^ Group comparisons performing using univariate Wilcoxon rank sum tests. *P-values < 0.05, **P ≤ 0.001. NPI = Neuropsychiatric Inventory.

Group comparisons between former rugby players and controls for self and informant completed symptom questionnaires are displayed in Table 2 and Fig. 2. Compared to control subjects, former players had evidence of worse quality of life scores, higher pain symptoms score, higher burden of depressive symptoms, higher burden of anxiety symptoms, higher burden of post-concussion symptoms defined by the mRPO (including ‘atypical’ concussion symptoms) and higher self-reported behaviour rating of executive dysfunction. Informant-reported questionnaires also revealed evidence of differences between controls and former players with higher scores of informant behaviour rating of executive dysfunction, neuropsychiatric symptom severity and neuropsychiatric symptom distress. Former players were also more likely to score above clinically significant thresholds than controls on questionnaires of self-rated depression symptoms, with 28.5% [95% confidence interval (CI) = 22.2–35.4%] of players scoring 14 points or higher on the BDI scale (signifying mild depression), compared to 3.1% (95% CI = 0.00–16.2%) of controls (χ^2^ = 8.49, df = 1, *P* = 0.004). Subjective memory complaints were common, with 55% of former players reporting at least mild problems on the memory component of the mRPO and 26% reporting at least moderate problems. There were no differences in sleep quality or insomnia measured by the Pittsburgh Sleep Quality Index and the ISI.

**Table 2:** Comparison of symptom-based questionnaires between former elite rugby players and non-sporting control group

Symptom based questionnaires	Questionnaire scores			Proportion above cut-off, *n*/*n* (%)		
	Former rugby player	Non-sporting control	*P*-value^a^	Former rugby player	Non-sporting control	*P*-value^b^
Self-reported questionnaire scores						
Quality of life (EuroQol 5dim), median (IQR)	7.0^c^ (5.0–8.0)	6.0 (5.0–7.0)	0.011*	–	–	–
Pain symptoms (EuroQol 5dim pain), median (IQR)	2.0^c^ (1.0–2.0)	1.0 (1.0–2.0)	0.027*	–	–	–
Depression symptoms (BDI), median (IQR)	8.0 (4.0–15.0)	5.5 (1.0–9.0)	0.011*	55/193 (28.5%)	1/32 (3.1%)	0.004*
Anxiety symptoms (GAD7), median (IQR)	3.0 (1.0–6.0)	1.0 (0.0–4.0)	0.001*	68/193 (35.2%)	7/33 (21.2%)	0.17
Total post-concussion symptoms (modified Rivermead), median (IQR)	14.0^d^ (7.0–25.0)	6.5^e^ (0.8–10.2)	<0.001*	–	–	–
Typical post-concussion symptoms (modified Rivermead), median (IQR)	13.0^d^ (7.0–22.0)	6.5^e^ (0.8–9.0)	<0.001*	–	–	–
Atypical post-concussion symptoms (modified Rivermead), median (IQR)	0.0^d^ (0.0–2.0)	0.0^e^ (0.0–0.0)	0.007*	–	–	–
Sleep quality index score (PSQI), median (IQR)	6.0 (4.0–9.0)	6.0 (4.0–7.0)	0.52	109/193 (56.5%)	19/33 (57.6%)	1.0
Insomnia severity (ISI), median (IQR)	6.0 (2.0–10.0)	5.0 (2.0–8.2)	0.46	76/192 (39.6%)	11/32 (34.4%)	0.72
Behaviour rating of executive dysfunction (BRIEF-A GEC), median (IQR)	50.0 (42.0–62.0)	42.5 (37.0–48.2)	<0.001*	27/191 (14.1%)	1/32 (3.1%)	0.15
Informant-reported questionnaire scores						
Behaviour rating of executive dysfunction (BRIEF-A GEC), median (IQR)	51.0 (43.2–57.0)	43.0 (41.0–48.0)	<0.001*	19/178 (10.7%)	0/31 (0%)	0.12
Neuropsychiatric symptom severity (NPIQ), median (IQR)	2.0^f^ (0.0–5.0)	0.0^g^ (0.0–1.0)	<0.001*	–	–	–
Neuropsychiatric symptom caregiver distress (NPIQ), median (IQR)	2.0^f^ (0.0–7.0)	0.0^g^ (0.0–1.0)	<0.001*	–	–	–

*n* = 200 former rugby players and 33 non-sporting control subjects, unless otherwise specified under ‘Proportion above cut-off'. Pre-existing clinically significant cut-offs were used for questionnaires where possible. Pre-existing cut-off scores: Becks Depression Inventory (BDI) ≥ 14; Generalised Anxiety Disorder 7 (GAD7) ≥ 5; Pittsburgh Sleep Quality Index (PSQI) ≥ 6; Insomnia Severity Index (ISI) ≥ 8; Behavioural Rating Inventory of Executive Function Adult (BRIEF-A) GEC ≥ 65. IQR = interquartile range; NPIQ = Neuropsychiatric Inventory Questionnaire.

^a^Wilcoxon rank sum test.

^b^Chi-squared test.

^c^ *n* = 192.

^d^ *n* = 191.

^e^ *n* = 32.

^f^ *n* = 167.

^g^ *n* = 30.

^*^ *P*-values < 0.05.

### Former rugby players have similar performance to healthy controls on formal cognitive testing

**Figure 3:**
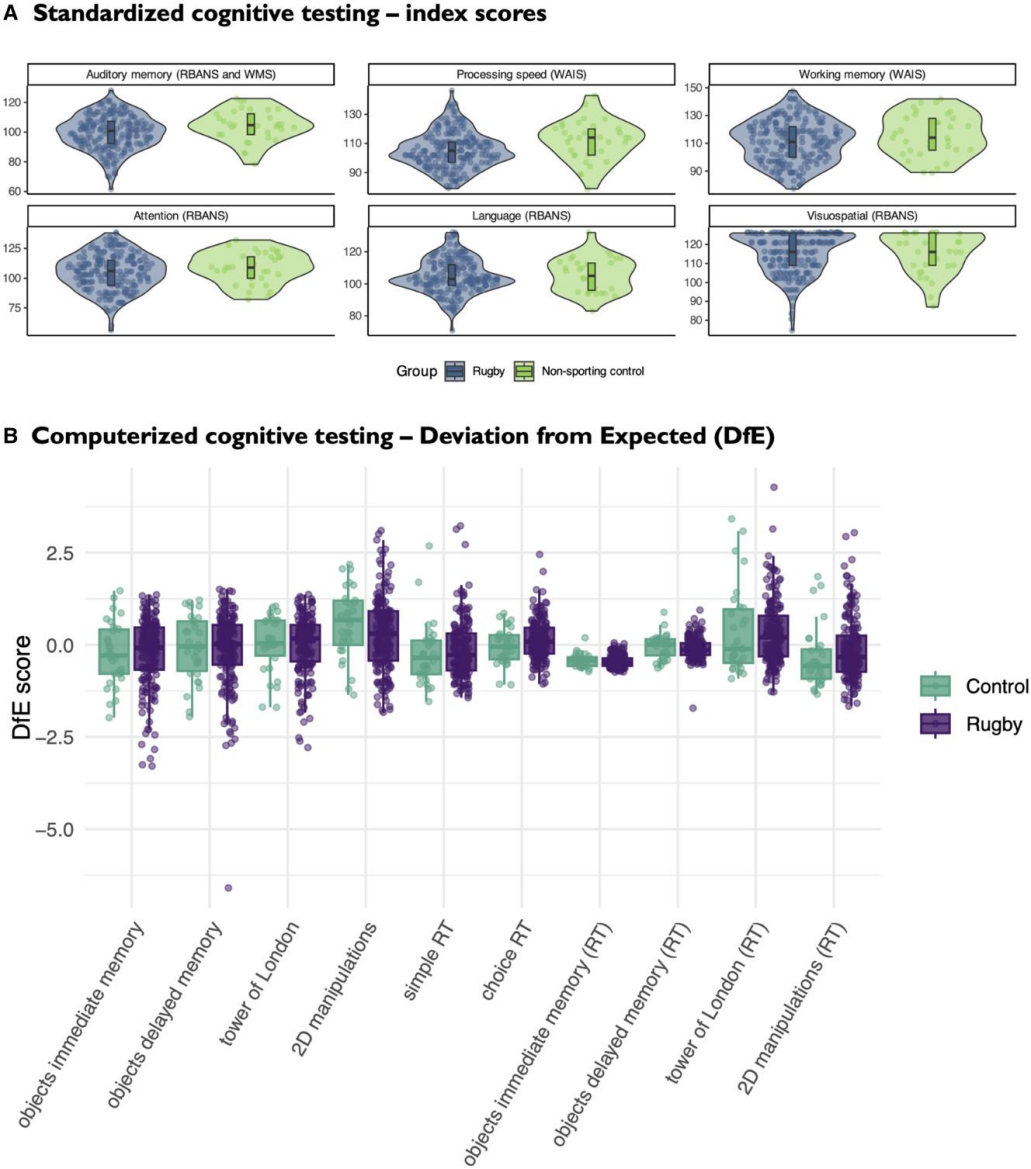
**Cognitive performance in former elite rugby players and healthy control subjects**. (**A**) Violin plots comparing index scores from Repeatable Battery for the Assessment of Neuropsychological Status (RBANS) and Wechsler Memory Scale (WMS) in both groups. (**B**) Performance on Cognitron tasks for both groups estimated against the large normative dataset using deviation from expected (DfE) scores. DfE scores derived from linear models trained to the normative dataset to predict each score based on a range of demographic factors (i.e. age, age squared, gender, education, language, handedness and device).^41,49^ Group comparisons were performed using general linear model. WAIS = Wechsler Adult Intelligence Scale.

Group comparisons between former players and controls for standardized tests of neuropsychology are displayed in Table 3 and Fig. 3A. Mean estimated premorbid functioning for the ex-players fell in the average range (105), whilst the mean of non-sporting controls was six points higher at 111 (*P* < 0.001) and fell within the high-average range. On all standardized neuropsychological tests of current cognitive functioning, there were no significant differences observed between former players and control subjects. Average index scores for former rugby players either fell within the normal range (auditory memory, processing speed and attention) or the high-average range (working memory and visuospatial function). Sixteen former players (8.1%) were defined as having objective auditory memory impairment (i.e. 1.5 SD below premorbid levels), but this proportion was not significantly different from the control group. The proportion of former players demonstrating objective evidence of impaired functioning in the other tests ranged from 0.5% (working memory) to 4.5% (executive function and attention) and again this was not different to the proportion observed in the control group. There were also no failures on the performance validity testing in the former players. There were also no differences in performance between groups on all computerized tests of cognition (Table 4 and Fig. 3B).

**Table 3:** Comparison of standardized neuropsychology tests between former elite rugby players and non-sporting control group.

Standardized neuropsychology	Test scores			Proportion impaired *n*/*n* (%)		
	Former rugby player	Non-sporting control	*P*-value^a^	Former rugby player	Non-sporting control	*P*-value^b^
Estimated premorbid functioning (FSIQ), mean (SD)	105 (8)^c^	111 (8)	<0.001*	–	–	–
Performance validity (Dot counting score), median (IQR)	9.5 (7.8–11.2)	9.7 (7.7–11.0)	0.12	3/199 (1.5%)	1/33 (3%)	0.37
Performance validity (Digit span, WAIS IV), median (IQR)	10.0 (9.0–12.0)	11.0 (9.0–12.0)	0.75	8/199 (4%)	3/33 (9.1%)	0.11
Executive function (Trail making test, B versus A ratio), median (IQR)	2.1^c^ (1.8–2.5)	2.0 (1.8–2.4)	0.78	–	–	–
Executive function (D-KEFS inhibition switching versus baseline contrast), median (IQR)	30.9^d^ (25.3–36.7)	31.2 (26.2–38.2)	0.62	–	–	–
Auditory memory composite score (RBANS and WMS IV), mean (SD)	99.6 (11.6)	104.5 (10.5)	0.29	16/198 (8.1%)	3/33 (9.1%)	0.56
Processing speed index score (WAIS IV), mean (SD)	105.5 (12.5)	111.7 (13.8)	0.22	8/199 (4%)	2/33 (6.1%)	0.67
Working memory index score (WAIS IV), mean (SD)	111.3 (15.1)	116.0 (15.3)	0.31	1/199 (0.5%)	0/33 (0%)	0.99
Attention index score (RBANS), mean (SD)	105.1 (15.4)	108.9 (13.2)	0.91	9/199 (4.5%)	3/33 (9.1%)	0.48
Language index score (RBANS), mean (SD)	104.7 (10.9)	105.5 (10.6)	0.9	3/199 (1.5%)	3/33 (9.1%)	0.053
Visuospatial index score (RBANS), mean (SD)	115.4 (10.5)	115.5 (10.8)	0.66	2/199 (1%)	1/33 (3%)	0.37

*n* = 200 former rugby players and 33 non-sporting control subjects, unless otherwise specified under ‘Proportion impaired'. Cognitive impairment on neuropsychology tasks were defined by an index score that is ≥1.5 SD below each individual's FSIQ. D-KEFS = Delis-Kaplan Executive Function System; FSIQ = Full-Scale Intelligence Quotient; IQR = interquartile range; RBANS = Repeatable Battery for the Assessment of Neuropsychological Status; SD = standard deviation; WAIS IV = Wechsler Adult Intelligence Scale, Fourth edition; WMS IV = Wechsler Memory Scale—Fourth edition.

^a^Linear regression adjusted for age, education years and sex.

^b^Logistic regression adjusted for age, education years and sex.

^c^ *n* = 199.

^d^ *n* = 198.

^*^ *P*-values < 0.05.

**Table 4:** Comparison of computerized neuropsychology tests between former elite rugby players and non-sporting control group.

Cognitron computerized neuropsychology	Raw scores			Deviation from expected score		
	Former rugby player	Non-sporting control	*P*-value	Former rugby player	Non-sporting control	*P*-value
Objects immediate memory accuracy, mean (SD)	48.2 (6.4)	49.5 (5.3)	0.48^a^	−0.23 (0.95)	−0.25 (0.87)	0.35^c^
Objects immediate memory duration (seconds), mean (SD)	160.1 (26.5)	162.6 (29.2)	0.39^b^	−0.44 (0.16)	−0.43 (0.18)	0.51^c^
Objects delayed memory accuracy, mean (SD)	47.1(6.5)	48.2 (6.3)	0.65^a^	−0.14(1.02)	−0.07 (0.90)	0.38^c^
Objects delayed memory duration (s), mean (SD)	82.0 (14.1)	85.9 (17.0)	0.18^b^	−0.11 (0.30)	−0.05 (0.34)	0.12^c^
Tower of London accuracy, mean (SD)	7.1(2.0)	7.3 (1.8)	0.80^a^	0.01(0.82)	0.08 (0.71)	0.91^c^
Tower of London RT (s), mean (SD)	215.5 (78.8)	13.6 (6.1)	0.50^b^	0.33 (0.91)	0.44 (1.17)	0.52^c^
2D Manipulations accuracy, mean (SD)	29.8 (7.9)	30.0 (8.0)	0.74^a^	0.33 (0.99)	0.58 (0.95)	0.90^c^
2D Manipulations RT (s), mean (SD)	4.5 (1.6)	4.5 (1.6)	0.96^b^	−0.17 (0.85)	−0.37 (0.83)	0.98^c^
Simple reaction time (ms), mean (SD)	318.2 (43.9)	323.8 (51.3)	0.72^b^	−0.16 (0.78)	−0.10 (0.84)	0.81^c^
Choice reaction time (ms), mean (SD)	482.7 (46.0)	483.7 (53.3)	0.99^b^	0.11 (0.57)	0.08 (0.71)	0.62^c^

*n* = 188 former rugby players and 33 non-sporting control subjects. RT = reaction time; SD = standard deviation.

^a^Linear regression adjusted for age, education years and sex.

^b^Generalized linear model with gamma log link function.

^c^Deviation from expected (DfE) scores derived from linear models trained to the normative dataset to predict each score based on a range of demographic factors (i.e. age, age squared, gender, education, language, handedness and device)^39,47^ group comparisons performed using general linear model.

### Dementia and traumatic encephalopathy syndrome

**Figure 4:**
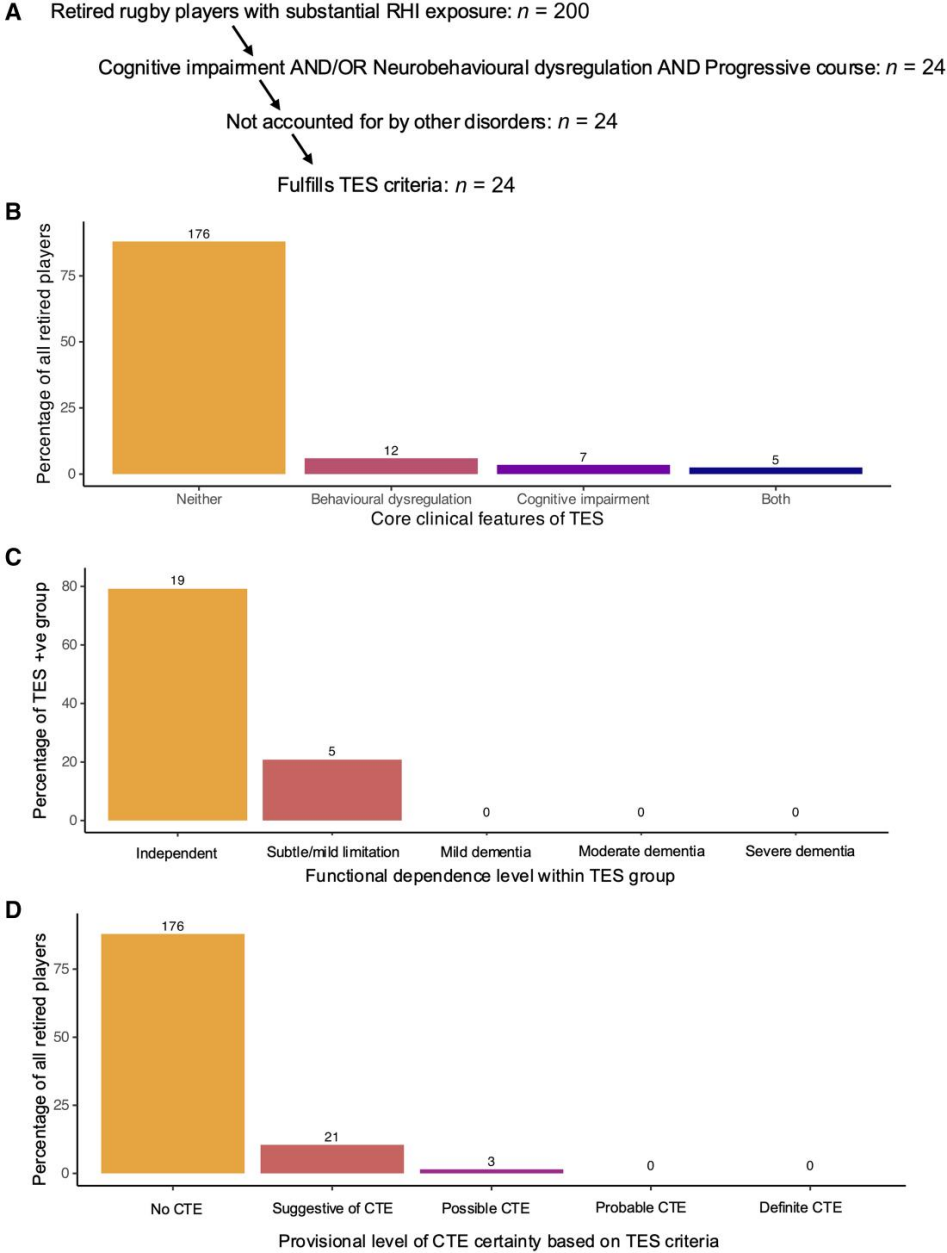
**Traumatic encephalopathy syndrome framework applied to 200 UK-based former rugby players.** ^20^ (**A**) Stepwise process to determine traumatic encephalopathy syndrome (TES) positivity. (**B**) Proportions of former players with progressive cognitive impairment, progressive neurobeahvioural dysregulation or both. (**C**) Levels of functional dependence with TES positive group. (**D**) Provisional levels of certainty for chronic traumatic encephalopathy (CTE) pathology. ‘Definite’ CTE requires pathological evidence. ‘Probable’ is the highest level of provisional certainty with pathological confirmation, whereas ‘suggestive’ is the lowest level of CTE provisional certainty. RHI = repetitive head impacts.

No participants fulfilled criteria for mild, moderate or severe dementia (Fig. 4). However, criteria for TES were fulfilled by 24/200 (12%) former players. Median age of TES positive individuals was 45 (IQR = 13) years and 22/24 were male (91.7%). Isolated cognitive impairment of a progressive nature was present in 7/200 (3.5%), whilst isolated progressive neurobehavioral dysregulation was present in 12/200 (6%). Both cognitive impairment and behavioural dysregulation were present in five former players (2.5%). None of the participants who underwent neurological examination had parkinsonian signs or abnormalities of the motor system. There were no instances where another disorder was found to fully account for their presentation. There was no association between TES and presence of a CSP (TES positive = 7/24; TES negative = 41/176; χ^2^ = 0.14, df = 1, *P* = 0.71). None of the TES positive cases had evidence of microhaemorrhages (TES positive = 0/24; TES negative = 6/176; χ^2^ = 0.89, df = 1, *P* = 0.34). In terms of provisional levels of certainty for CTE pathology proposed by the TES framework, 0/24 former players were deemed to have probable CTE (probable being the highest level of certainty possible in participants without post-mortem data), 3/24 were deemed to have a clinical phenotype consistent with ‘possible’ CTE pathology, while 21/24 former players were deemed to have a clinical phenotype ‘suggestive’ of CTE pathology (suggestive being the least certain). In terms of functional dependence level, 19 of the TES participants were deemed to be functionally independent and five participants were deemed to have mild functional limitation.

### Associations of self-reported concussion

Demographic characteristics of former players self-reporting low and high numbers of career concussions (based on a median split of seven) are displayed in Supplementary Table 1. Former players in the high concussion group were heavier, taller, had greater muscle mass, were more likely to be forwards rather than backs, had higher scores related to problematic drinking on the AUDIT questionnaire and more likely to report illicit drug use. The high-concussion group also had a higher proportion of individuals with a CSP grade 2 or above (32% versus 16%, *P* = 0.014).

Symptom burden was elevated in the high concussion group compared to the low concussion group with differences in depression symptoms, anxiety symptoms, typical post-concussion symptoms, ‘atypical’ post-concussion symptoms, self-reported and informant reported behaviour rating of executive dysfunction, and informant reported neuropsychiatric symptom severity (see Supplementary Table 2 for full results). There were no statistically significant differences on standardized or computerized tests of cognition between high and low concussion groups following correction for multiple comparisons (Supplementary Tables 3 and 4). Sixteen of 97 of the former players in the high-concussion group were defined as TES compared to 7/101 in the low concussion group (χ^2^ = 3.52, df = 1, *P* = 0.06). The high-concussion group had a higher proportion of individuals defined as TES positive based on progressive neurobehavioural disturbance: 13/97 compared to 3/101 in the low-concussion group (χ^2^ = 5.91, df = 1, *P* = 0.015). There was no difference in the number of former players defined as TES positive because of progressive cognitive impairment: 8/97 in high concussion group, 4/101 in the low concussion group (χ^2^ = 0.93, df = 1, *P* = 0.33).

### Associations with career duration

The relationship between career length and former player demographic characteristics is displayed in Table 1. There was a positive correlation between career length and age at assessment, body weight, and body mass index. Males had longer career durations than females. Longer career duration was negatively correlated with years of education. Those reporting illicit drug use had shorter careers than those with no reported illicit drug use. There were no statistically significant relationships observed between career duration and symptom burden (Supplementary Table 5). There were no associations observed between career length and performance on standardized tests of cognition (Supplementary Table 6), or computerized tests of cognition (Supplementary Table 7). Median career duration for players defined as TES positive was 11 years (IQR = 6.25) and for TES negative was 10.5 years (IQR = 5). This was not significantly different (*P* = 0.7). This was also the case when subgrouping TES positive cases into those with progressive neurobehavioural disturbance (*P* = 0.66) or progressive cognitive impairment (*P* = 0.80).

### Associations of player position

Demographic characteristics of forwards and backs are displayed in Supplementary Table 8. Forwards had higher levels of self-reported concussions, were more likely to have white ethnic background, had greater body mass indices, greater height and weight, higher body fat percentages and higher muscle mass. Forwards had higher scores related to typical post-concussion symptoms and scored higher on a self-reported behaviour rating of executive dysfunction (Supplementary Table 9). There was no evidence of statistically significant differences in standardized tests of cognition between forwards and backs (Supplementary Table 10), but there was evidence that backs demonstrated quicker choice and simple reaction times on computerized testing compared to forwards (Supplementary Table 11). Eighteen of 126 forwards were defined as TES compared to 6/74 backs (χ^2^ = 1.15, df = 1, *P* = 0.28). Forwards were more likely to be TES positive than backs on the basis of progressive neurobehavioural disturbance, 15/126 compared to backs 2/74 (χ^2^ = 3.96, df = 1, *P* = 0.046). There was no difference in the number of players defined as TES positive based on progressive cognitive impairment; 8/126 in forwards and 4/74 in backs (χ^2^ = 0.00, df = 1, *P* = 1.00).

## Discussion

The consequence of a career in a contact sport such as rugby for brain health has become a major societal issue. However, the long-term effects of RHI exposure are unclear. In this study of 200 former elite rugby players in mid-life, we found a significant burden of cognitive and psychiatric symptoms, but relatively lower rates of objective cognitive impairment and no evidence of dementia. Depression and anxiety symptoms were relatively common and 12% of our cohort fulfilled research criteria for TES, but the pathological significance of this clinical classification remains uncertain. The results provide an important clinical context to better understand the relationship between elite rugby participation and brain health at mid-life.

The former players we studied all had significant RHI exposures during careers, which on average lasted just over 10 years. Previous work has shown career length to be one the strongest predictors of RHI-associated neuropathology and high numbers of self-reported concussions were common in our cohort.^19^ Recent analysis of instrumented mouth guard data shows significant head acceleration events are more common in forwards than backs and in keeping with this observation, we found self-reported concussions were higher in forwards than backs.^48^

Elite rugby participation has been associated with increased risk of neurodegenerative disease, but RHI exposure has also been linked to elevated risk of dementia and cognitive impairment in other sporting contexts, including American football and association football (soccer).^50-54^ Many of our former players reported significant concerns about their cognitive function, with subjective memory complaints frequently reported. Despite the high frequency of subjective cognitive problems, at the group level we did not find evidence of impaired cognition on objective tests when former players were compared to control subjects. In general, former players performed within the normal range of standardized cognitive tests. Similar findings have been observed in retired former elite male rugby league players in Australia.^55^ A minority scored lower than pre-morbid expectations, with 6% of players fulfilling TES criteria for cognitive impairment. We found no evidence of dementia i.e. significant disability as a result of the cognitive impairments in our cohort, although our sample may not be representative of all former rugby players, therefore the exact prevalence of dementia in this age group of former players is difficult to determine. It is important to note that at a general population level, dementia incidence and prevalence in the age range examined (i.e. mid-life) is very low and tends to exponentially increase from the seventh decade onward, so does not preclude an increased risk of dementia from rugby participation later in older age ranges.^56^

More generally, former players reported a wide range of symptoms on most scales except sleep quality. High rates of psychiatric symptoms have previously been reported in former athletes with significant exposure to RHI.^57,58^ Consistent with this, 28.5% and 35.2% of our former players had abnormally high levels of depressive and anxiety symptoms, respectively rising to almost 38.9% and 42.2% in those with high numbers of concussions, both of which are higher than population estimates from general population datasets.^59^ These results show that psychiatric complaints are common in former rugby players and should be an important focus when clinically assessing former elite athletes exposed to RHI.^60^ A caveat in the interpretation of the high rate of psychiatric symptoms is that there is likely an ascertainment bias towards former players with higher symptoms in a brain health clinic setting, which is supported by data highlighting that symptoms of depression are common in individuals seen in memory clinics more generally.^61^ Furthermore, scores for atypical symptoms embedded within the Rivermead Post-concussion Symptoms Questionnaire were higher in former players, especially the high-concussion group, suggesting that hypervigilance may be a factor in their clinical presentation.^62,63^

MRI was performed in almost all former players, but we found little evidence for TBI or extensive neurodegeneration. Only two ex-players had evidence of brain atrophy thought to be greater than expected for normal age on visual clinical radiology reporting. The most striking MRI finding was the presence of CSP in almost one-quarter of ex-players, which is defined by the presence of CSF between the septal leaflets, which separate the frontal horns of the lateral ventricles.^64^ Furthermore, CSP was twice as likely to be seen in ex-players from the high concussion group compared to the low concussion group (32% versus 16%). CSP is more common in individuals exposed to RHI and neuropathological studies have suggested an increased likelihood of CTE pathology in individuals with evidence of CSP.^7,9,64,65^ This may reflect the cumulative exposure to head impacts, which could explain an association with CTE pathology as high biomechnical strain, particularly observed at the base of the brain's sulci, is seen at locations of perivascular tau pathology characteristic of CTE.^66^ We also found a relatively small number of other post-traumatic abnormalities: 3% (6/199) of the ex-players had microbleeds, an imaging marker of traumatic vascular injury, which is a slightly lower percentage than we previously reported in active elite rugby players (7%, 3/44).^5,67^

An important question is whether RHI exposure initiates or accelerates neurodegenerative processes that may have not yet affected cognitive performance, but that might cause impairments in later life. There are a number of potential mechanisms for this, including initiating or accelerating the accumulation of tau, amyloid, synuclein and TDP-43 pathologies.^7,68^ Neurodegenerative diseases, such as Alzheimer's disease, frontotemporal lobar degeneration and CTE have long prodromal phases, where progressive pathology is present without clinical symptoms of dementia.^69-71^ If RHI accelerates neurodegenerative processes this might increase the dementia risk or bring forward the age at which it manifests.^56^

CTE has been reported in large numbers of American football players, as well as smaller groups of former rugby players across a wide range of ages, including as young as 23 years old.^11,14,16,18-20^ However, the clinical effects and natural history of this pathology is unclear. Of our population, 12% fulfilled the TES criteria, with former players who were forwards or who reported high number of concussions more likely to be classified as having TES based on neurobehavioural disturbance (although not cognitive impairment). In contrast, TES classification was not associated with long career duration, which has previously been linked to CTE pathology.^10,12,19^ Interpretating this classification is challenging. First, it is made on clinical grounds, with relatively non-specific contributing symptoms. Progression of clinical symptoms is a key component of the TES criteria. Given the cross-sectional nature of this analysis, it is very difficult to assess progression without repeated assessment and we relied on subjective aspects of the clinical history rather than objective decline from serial assessment. Longitudinal study of this cohort will be vital to further clarify this issue. Furthermore, the levels of certainty for CTE pathology based on criteria within the TES framework were low, with the majority of ex-players classified as having a phenotype suggestive of CTE (the lowest level of provisional certainty). Finally, the TES criteria are largely based on retrospective analysis of post-mortem cases of American footballers and may be less applicable to other sporting contexts.^72^ Application of the TES criteria to a similarly aged cohort of boxers and mixed martial arts fighters found higher rates of TES (41%).^73^ Whether this is related to different risk profiles in individual sports or differential application of the TES criteria is unclear. Ultimately, biomarker confirmation will be necessary to confirm the presence of relevant CTE pathologies and long-term follow-up is needed to understand whether a TES classification in mid-life increases the risk of later dementia or other neurobehavioural disabilities.

Alcohol consumption was relatively high in our cohort, which may contribute to behavioural, psychiatric and cognitive symptoms.^74^ Excess alcohol is a modifiable risk factor for dementia and is linked to deleterious effects on brain structure, including in former rugby players, highlighting the importance of pro-active screening and management of alcohol misuse in individuals exposed to RHI.^75-77^ A further important factor contributing to difficulties experienced by our former players are the challenges to adjusting to life after retirement.^78,79^ This is particularly likely to influence the mental health of former athletes and is a potential cause of a range of the symptoms we observed.

One limitation of our study is that the group of former players studied may not be entirely representative of the former rugby player population more broadly. Although the study population consisted of a wide range of former players in terms of position and head impact exposure, the single location of the study (London, UK) could make attendance for those with severe functional impairment due to neurodegeneration less likely to attend. However, there were no individuals who expressed interest in participating in the study that were not able to attend in person. In addition, a concurrent legal action against the sport's governing bodies may also have led to some players with significant issues not participating as they had already been assessed as part of the legal proceedings. In addition, there was a low proportion of female participants recruited, meaning we are likely underpowered to investigate sex-specific effects. A further limitation is the accuracy of retrospective assessments of head impact exposure. Self-reported estimates are imprecise, and not all symptoms related to concussion are necessarily related to TBI. In the future, more accurate and objective measurement of head impact exposure from tools, such as instrumented mouthguards and integration of biomarkers related to brain injury during player's careers, will enable more certainty when relating outcomes to head impact exposure sustained during a rugby career.^2,5,6,48,77^

In conclusion, in this baseline assessment of 200 former rugby players in mid-life with brain health concerns and high rates of subjective cognitive complaints, we found evidence of significant psychiatric symptom burden, which was related to number of self-reported career concussions. In contrast, evidence of objective cognitive impairments and TES were uncommon and there was no evidence of dementia.

## Supplementary Material

awae416_Supplementary_Data

## Data Availability

Following completion of data collection and primary publications, data will be made available upon reasonable request from *bona fide* researchers.

## References

[1] Cross M, Kemp S, Smith A, Trewartha G, Stokes K. Professional rugby union players have a 60% greater risk of time loss injury after concussion: A 2-season prospective study of clinical outcomes. Br J Sports Med. 2016;50:926–931. 26626266 10.1136/bjsports-2015-094982 PMC4975843

[2] Laverse E, Guo T, Zimmerman K, et al Plasma glial fibrillary acidic protein and neurofilament light chain, but not tau, are biomarkers of sports-related mild traumatic brain injury. Brain Commun. 2020;2:fcaa137. 33543129 10.1093/braincomms/fcaa137 PMC7846133

[3] Russell ER, Mackay DF, Lyall D, et al Neurodegenerative disease risk among former international rugby union players. J Neurol Neurosurg Psychiatry. 2022;93:1262–1268. 36195436 10.1136/jnnp-2022-329675 PMC9669247

[4] Gallo V, McElvenny DM, Seghezzo G, et al Concussion and long-term cognitive function among rugby players-The BRAIN Study. Alzheimers Dement. 2022;18:1164–1176. 34668650 10.1002/alz.12455 PMC9298292

[5] Zimmerman KA, Laverse E, Samra R, et al White matter abnormalities in active elite adult rugby players. Brain Commun. 2021;3:fcab133. 34435188 10.1093/braincomms/fcab133 PMC8381344

[6] Parker TD, Zimmerman KA, Laverse E, et al Active elite rugby participation is associated with altered precentral cortical thickness. Brain Commun. 2023;5:fcad257. 38025272 10.1093/braincomms/fcad257 PMC10667029

[7] Smith DH, Johnson VE, Trojanowski JQ, Stewart W. Chronic traumatic encephalopathy—Confusion and controversies. Nat Rev Neurol. 2019; 15:179–183. 30664683 10.1038/s41582-018-0114-8 PMC6532781

[8] McKee AC, Stein TD, Huber BR, et al Chronic traumatic encephalopathy (CTE): Criteria for neuropathological diagnosis and relationship to repetitive head impacts. Acta Neuropathol. 2023;145:371–394. 36759368 10.1007/s00401-023-02540-w PMC10020327

[9] McKee AC, Stern RA, Nowinski CJ, et al The spectrum of disease in chronic traumatic encephalopathy. Brain. 2013;136(Pt 1):43–64. 23208308 10.1093/brain/aws307 PMC3624697

[10] Mez J, Daneshvar DH, Abdolmohammadi B, et al Duration of American football play and chronic traumatic encephalopathy. Ann Neurol. 2020;87:116–131. 31589352 10.1002/ana.25611 PMC6973077

[11] Mez J, Daneshvar DH, Kiernan PT, et al Clinicopathological evaluation of chronic traumatic encephalopathy in players of American football. JAMA. 2017;318:360–370. 28742910 10.1001/jama.2017.8334 PMC5807097

[12] Daneshvar DH, Nair ES, Baucom ZH, et al Leveraging football accelerometer data to quantify associations between repetitive head impacts and chronic traumatic encephalopathy in males. Nat Commun. 2023;14:3470. 37340004 10.1038/s41467-023-39183-0 PMC10281995

[13] Alosco ML, Mian AZ, Buch K, et al Structural MRI profiles and tau correlates of atrophy in autopsy-confirmed CTE. Alzheimers Res Ther. 2021;13:193. 34876229 10.1186/s13195-021-00928-y PMC8653514

[14] Stewart W, McNamara PH, Lawlor B, Hutchinson S, Farrell M. Chronic traumatic encephalopathy: A potential late and under recognized consequence of rugby union? QJM. 2016;109:11–15. 25998165 10.1093/qjmed/hcv070

[15] Buckland ME, Sy J, Szentmariay I, et al Chronic traumatic encephalopathy in two former Australian National Rugby League players. Acta Neuropathol Commun. 2019;7:97. 31242928 10.1186/s40478-019-0751-1 PMC6595631

[16] Lee EB, Kinch K, Johnson VE, Trojanowski JQ, Smith DH, Stewart W. Chronic traumatic encephalopathy is a common co-morbidity, but less frequent primary dementia in former soccer and rugby players. Acta Neuropathol. 2019;138:389–399. 31152201 10.1007/s00401-019-02030-y PMC6689293

[17] Stein TD, Montenigro PH, Alvarez VE, et al Beta-amyloid deposition in chronic traumatic encephalopathy. Acta Neuropathol. 2015;130:21–34. 25943889 10.1007/s00401-015-1435-y PMC4529056

[18] Lee EB, Kennedy-Dietrich C, Geddes JF, et al The perils of contact sport: Pathologies of diffuse brain swelling and chronic traumatic encephalopathy neuropathologic change in a 23-year-old rugby union player. Acta Neuropathol. 2023;145:847–850. 37086326 10.1007/s00401-023-02576-y PMC10175208

[19] Stewart W, Buckland ME, Abdolmohammadi B, et al Risk of chronic traumatic encephalopathy in rugby union is associated with length of playing career. Acta Neuropathol. 2023;146:829–832. 37872234 10.1007/s00401-023-02644-3 PMC10627955

[20] Katz DI, Bernick C, Dodick DW, et al National institute of neurological disorders and stroke consensus diagnostic criteria for traumatic encephalopathy syndrome. Neurology. 2021;96:848–863. 33722990 10.1212/WNL.0000000000011850 PMC8166432

[21] Zimmerman KA, Hain JA, Graham NSA, et al A prospective cohort study of long-term neurological outcomes in retired elite athletes: The advanced BiomaRker, Advanced Imaging and Neurocognitive (BRAIN) Health Study protocol. BMJ Open. 2024;14:e082902. 10.1136/bmjopen-2023-082902 PMC11043776 38663922

[22] James L, Davies M, Mian S, et al The BRAIN-Q, a tool for assessing self-reported sport-related concussions for epidemiological studies. Epidemiol Health. 2021;43:e2021086. 34696571 10.4178/epih.e2021086 PMC8863616

[23] Corrigan JD, Bogner J. Initial reliability and validity of the Ohio state university TBI identification method. J Head Trauma Rehabil. 2007;22:318–329. 18025964 10.1097/01.HTR.0000300227.67748.77

[24] Saunders JB, Aasland OG, Babor TF, de la Fuente JR, Grant M. Development of the Alcohol Use Disorders Identification Test (AUDIT): WHO Collaborative Project on early detection of persons with harmful alcohol consumption—II. Addiction. 1993;88:791–804. 8329970 10.1111/j.1360-0443.1993.tb02093.x

[25] Berman AH, Bergman H, Palmstierna T, Schlyter F. Evaluation of the Drug Use Disorders Identification Test (DUDIT) in criminal justice and detoxification settings and in a Swedish population sample. Eur Addict Res. 2005;11:22–31. 15608468 10.1159/000081413

[26] Beck AT, Ward CH, Mendelson M, Mock J, Erbaugh J. An inventory for measuring depression. Arch Gen Psychiatry. 1961;4:561–571. 13688369 10.1001/archpsyc.1961.01710120031004

[27] Spitzer RL, Kroenke K, Williams JBW, Löwe B. A brief measure for assessing generalized anxiety disorder: The GAD-7. Arch Intern Med. 2006;166:1092–1097. 16717171 10.1001/archinte.166.10.1092

[28] Bastien CH, Vallières A, Morin CM. Validation of the insomnia severity index as an outcome measure for insomnia research. Sleep Med. 2001;2:297–307. 11438246 10.1016/s1389-9457(00)00065-4

[29] Buysse DJ, Reynolds CF, Monk TH, Berman SR, Kupfer DJ. The Pittsburgh sleep quality index: A new instrument for psychiatric practice and research. Psychiatry Res. 1989;28:193–213. 2748771 10.1016/0165-1781(89)90047-4

[30] Brooks KJL, Sullivan KA. Validating the modified Rivermead post-concussion symptoms questionnaire (mRPQ). Clin Neuropsychol. 2023;37:207–226. 34348079 10.1080/13854046.2021.1942555

[31] Herdman M, Gudex C, Lloyd A, et al Development and preliminary testing of the new five-level version of EQ-5D (EQ-5D-5L). Qual Life Res. 2011;20:1727–1736. 21479777 10.1007/s11136-011-9903-x PMC3220807

[32] Roth RM, Isquith PK, Gioia GA. Behavior rating inventory of executive function®—Adult version. APA PsycTests. 2005. Accessed 10 April 2025. doi:10.1037/t86244-000

[33] Cummings JL . The neuropsychiatric inventory: Assessing psychopathology in dementia patients. Neurology. 1997;48(5 Suppl 6):S10–S16. 10.1212/wnl.48.5_suppl_6.10s 9153155

[34] Wechsler D . Wechsler Adult Intelligence Scale. Fourth Edition. APA PsycTests. 2008. Accessed 10 April 2025. doi:10.1037/t15169-000

[35] Boone KB, Lu P, Back C, et al Sensitivity and specificity of the Rey dot counting test in patients with suspect effort and various clinical samples. Arch Clin Neuropsychol. 2002;17:625–642. 14591847

[36] Delis DC, Kaplan E, Kramer JH. Delis-Kaplan executive function system. APA PsycTests. 2001. Accessed 10 April 2025. doi:10.1037/t15082-000

[37] Tombaugh TN . Trail making test A and B: Normative data stratified by age and education. Arch Clin Neuropsychol. 2004;19:203–214. 15010086 10.1016/S0887-6177(03)00039-8

[38] Wechsler D . Wechsler Memory Scale. Fourth Edition. APA PsycTests. 2009. Accessed 10 April 2025. doi: 10.1037/t15175-000

[39] Randolph C, Tierney MC, Mohr E, Chase TN. The repeatable battery for the assessment of neuropsychological status (RBANS): Preliminary clinical validity. J Clin Exp Neuropsychol. 1998;20:310–319. 9845158 10.1076/jcen.20.3.310.823

[40] Jolly AE, Scott GT, Sharp DJ, Hampshire AH. Distinct patterns of structural damage underlie working memory and reasoning deficits after traumatic brain injury. Brain. 2020;143:1158–1176. 32243506 10.1093/brain/awaa067 PMC7174032

[41] Giovane MD, Trender WR, Bălăeţ M, et al Computerised cognitive assessment in patients with traumatic brain injury: An observational study of feasibility and sensitivity relative to established clinical scales. EClinicalMedicine. 2023;59:101980. 37152359 10.1016/j.eclinm.2023.101980 PMC10154960

[42] Hampshire A, Azor A, Atchison C, et al Cognition and memory after COVID-19 in a large community sample. N Engl J Med. 2024;390:806–818. 38416429 10.1056/NEJMoa2311330 PMC7615803

[43] Shallice T . Specific impairments of planning. Philos Trans R Soc Lond B Biol Sci. 1982;298:199–209. 6125971 10.1098/rstb.1982.0082

[44] Gardner RC, Hess CP, Brus-Ramer M, et al Cavum septum Pellucidum in retired American pro-football players. J Neurotrauma. 2016;33:157–161. 25970145 10.1089/neu.2014.3805 PMC4696427

[45] Brazier J, Antrobus M, Stebbings GK, et al Anthropometric and physiological characteristics of elite male rugby athletes. J Strength Cond Res. 2020;34:1790–1801. 30138238 10.1519/JSC.0000000000002827

[46] Cousins BEW, Morris JG, Sunderland C, Bennett AM, Shahtahmassebi G, Cooper SB. Training and match demands of elite rugby union. J Strength Cond Res. 2023;37:141–148. 36515599 10.1519/JSC.0000000000004237

[47] Paul L, Naughton M, Jones B, et al Quantifying collision frequency and intensity in rugby union and rugby sevens: A systematic review. Sports Med Open. 2022;8:12. 35050440 10.1186/s40798-021-00398-4 PMC8776953

[48] Tooby J, Woodward J, Tucker R, et al Instrumented mouthguards in elite-level men’s and women’s rugby union: The incidence and propensity of head acceleration events in matches. Sports Med. 2024;54:1327–1338. 37906425 10.1007/s40279-023-01953-7 PMC11127838

[49] Hampshire A, Trender W, Chamberlain SR, et al Cognitive deficits in people who have recovered from COVID-19. EClinicalMedicine. 2021;39:101044. 34316551 10.1016/j.eclinm.2021.101044 PMC8298139

[50] Mackay DF, Russell ER, Stewart K, MacLean JA, Pell JP, Stewart W. Neurodegenerative disease mortality among former professional soccer players. N Engl J Med. 2019;381:1801–1808. 31633894 10.1056/NEJMoa1908483 PMC8747032

[51] Walton SR, Brett BL, Chandran A, et al Mild cognitive impairment and dementia reported by former professional football players over 50 year of age: An NFL-LONG study. Med Sci Sports Exerc. 2022;54:424–431. 34593716 10.1249/MSS.0000000000002802

[52] Montenigro PH, Alosco ML, Martin BM, et al Cumulative head impact exposure predicts later-life depression, apathy, executive dysfunction, and cognitive impairment in former high school and college football players. J Neurotrauma. 2017;34:328–340. 27029716 10.1089/neu.2016.4413 PMC5220530

[53] Guskiewicz KM, Marshall SW, Bailes J, et al Association between recurrent concussion and late-life cognitive impairment in retired professional football players. Neurosurgery. 2005;57:719–726; discussion 719–726. 16239884 10.1093/neurosurgery/57.4.719

[54] Russell ER, Mackay DF, Stewart K, MacLean JA, Pell JP, Stewart W. Association of field position and career length with risk of neurodegenerative disease in male former professional soccer players. JAMA Neurol. 2021;78:1057–1063. 34338724 10.1001/jamaneurol.2021.2403 PMC8329793

[55] Van Patten R, Iverson GL, Terry DP, Levi CR, Gardner AJ. Predictors and correlates of perceived cognitive decline in retired professional rugby league players. Front Neurol. 2021;12:676762. 34707552 10.3389/fneur.2021.676762 PMC8542796

[56] Prince M, Prina M, Maëlenn G. World Alzheimer Report 2013: Journey of Caring: An analysis of long-term care for dementia. Published online 21 September 2013. Accessed 25 March 2024. https://www.alzint.org/resource/world-alzheimer-report-2013/

[57] Iverson GL, Van Patten R, Terry DP, Levi CR, Gardner AJ. Predictors and correlates of depression in retired elite level rugby league players. Front Neurol. 2021;12:655746. 33868156 10.3389/fneur.2021.655746 PMC8047059

[58] Roberts AL, Pascual-Leone A, Speizer FE, et al Exposure to American Football and Neuropsychiatric Health in former national football league players: Findings from the football players health study. Am J Sports Med. 2019;47:2871–2880. 31468987 10.1177/0363546519868989 PMC7163246

[59] Arias de la Torre J, Vilagut G, Ronaldson A, et al Prevalence and age patterns of depression in the United Kingdom. A population-based study. J Affect Disord. 2021;279:164–172. 33059219 10.1016/j.jad.2020.09.129

[60] Malhi GS, Mann JJ. Depression. Lancet. 2018;392:2299–2312. 30396512 10.1016/S0140-6736(18)31948-2

[61] Loreto F, Fitzgerald A, Golemme M, et al Prevalence of depressive symptoms in a memory clinic cohort: A retrospective study. J Alzheimers Dis. 2022;88:1179–1187. 35754270 10.3233/JAD-220170

[62] Windle K, Sullivan KA. Towards an embedded symptom validity indicator for the rivermead postconcussion symptom questionnaire. Appl Neuropsychol Adult. 2021;28:512–524. 34380355 10.1080/23279095.2019.1660880

[63] Clark CN, Edwards MJ, Ong BE, et al Reframing postconcussional syndrome as an interface disorder of neurology, psychiatry and psychology. Brain. 2022;145:1906–1915. 35472071 10.1093/brain/awac149 PMC9246708

[64] Asken BM, Rabinovici GD. Identifying degenerative effects of repetitive head trauma with neuroimaging: A clinically-oriented review. Acta Neuropathol Commun. 2021;9:96. 34022959 10.1186/s40478-021-01197-4 PMC8141132

[65] Stanwell P, Iverson GL, Patten RV, Castellani RJ, McCrory P, Gardner AJ. Examining for Cavum septum Pellucidum and ventricular enlargement in retired elite-level rugby league players. Front Neurol. 2022;13:817709. 35493804 10.3389/fneur.2022.817709 PMC9044485

[66] Ghajari M, Hellyer PJ, Sharp DJ. Computational modelling of traumatic brain injury predicts the location of chronic traumatic encephalopathy pathology. Brain. 2017;140:333–343. 28043957 10.1093/brain/aww317 PMC5278309

[67] Griffin AD, Turtzo LC, Parikh GY, et al Traumatic microbleeds suggest vascular injury and predict disability in traumatic brain injury. Brain. 2019;142:3550–3564. 31608359 10.1093/brain/awz290 PMC6821371

[68] Graham NS, Sharp DJ. Understanding neurodegeneration after traumatic brain injury: From mechanisms to clinical trials in dementia. J Neurol Neurosurg Psychiatry. 2019;90:1221–1233. 31542723 10.1136/jnnp-2017-317557 PMC6860906

[69] Rohrer JD, Nicholas JM, Cash DM, et al Presymptomatic cognitive and neuroanatomical changes in genetic frontotemporal dementia in the Genetic Frontotemporal dementia Initiative (GENFI) study: A cross-sectional analysis. Lancet Neurol. 2015;14:253–262. 25662776 10.1016/S1474-4422(14)70324-2 PMC6742501

[70] Bateman RJ, Xiong C, Benzinger TLS, et al Clinical and biomarker changes in dominantly inherited Alzheimer’s disease. N Engl J Med. 2012;367:795–804. 22784036 10.1056/NEJMoa1202753 PMC3474597

[71] Jia J, Ning Y, Chen M, et al Biomarker changes during 20 years preceding Alzheimer’s disease. N Engl J Med. 2024;390:712–722. 38381674 10.1056/NEJMoa2310168

[72] Asken BM, Tanner JA, VandeVrede L, et al Multi-modal biomarkers of repetitive head impacts and traumatic encephalopathy syndrome: A clinicopathological case series. J Neurotrauma. 2022;39:1195–1213. 35481808 10.1089/neu.2022.0060 PMC9422800

[73] Ritter A, Shan G, Montes A, Randall R, Bernick C. Traumatic encephalopathy syndrome: Application of new criteria to a cohort exposed to repetitive head impacts. Br J Sports Med. 2023;57:389–394. 36517216 10.1136/bjsports-2022-105819 PMC10086298

[74] McHugh RK, Weiss RD. Alcohol use disorder and depressive disorders. Alcohol Res. 2019;40:arcr.v40.1.01. 31649834 10.35946/arcr.v40.1.01 PMC6799954

[75] Livingston G, Huntley J, Sommerlad A, et al Dementia prevention, intervention, and care: 2020 report of the lancet commission. Lancet. 2020;396:413–446. 32738937 10.1016/S0140-6736(20)30367-6 PMC7392084

[76] Daviet R, Aydogan G, Jagannathan K, et al Associations between alcohol consumption and gray and white matter volumes in the UK biobank. Nat Commun. 2022;13:1175. 35246521 10.1038/s41467-022-28735-5 PMC8897479

[77] Wojtowicz M, Gardner AJ, Stanwell P, Zafonte R, Dickerson BC, Iverson GL. Cortical thickness and subcortical brain volumes in professional rugby league players. Neuroimage Clin. 2018;18:377–381. 29487794 10.1016/j.nicl.2018.01.005 PMC5814377

[78] Mannes ZL, Waxenberg LB, Cottler LB, et al Prevalence and correlates of psychological distress among retired elite athletes: A systematic review. Int Rev Sport Exerc Psychol. 2019;12:265–294. 31217807 10.1080/1750984X.2018.1469162 PMC6583001

[79] Park S, Lavallee D, Tod D. Athletes’ career transition out of sport: A systematic review. Int Rev Sport Exerc Psychol. 2013;6:22–53.

