# Supplementary material for: Brain health concerns in former rugby players: clinical and cognitive phenotypes
Source: Brain. 2025 Jul 3;148(8):2698–713. doi: 10.1093/brain/awae416 (PMC12316010; doi:10.1093/brain/awae416)
Supplement: awae416_Supplementary_Data [file awae416_supplementary_data.pdf]

	Low concussion (n=101)	High concussion (n=97)	p-value
Age, years, median (IQR)	45.0 (40.0 to 51.0)	43.0 (37.0 to 50.0)	0.35[a]
Male sex, *n* (%)	87 (86.1)	92 (94.8)	0.066[c]
Years of education, median (IQR)	16.0 (15.0 to 17.0)	16.0 (13.0 to 17.0)	0.094[a]
Ethnicity, *n* (%)			0.091[a]
White	92 (91.1)	96 (99.0)	
Black Caribbean	1 (1.0)	0 (0.0)	
Black African	3 (3.0)	0 (0.0)	
Other ethnic group (including 'mixed')	4 (4.0)	0 (0.0)	
Not stated	1 (1.0)	1 (1.0)	
Weight, kg, median (IQR)	97.9 (88.8 to 110.9)	104.1 (94.0 to 113.9)	**0.049*[a]**
Height, cm, mean (SD)	183.8 (9.2)	187.0 (8.7)	**0.015*[d]**
BMI, median (IQR)	28.8 (26.8 to 32.7)	29.3 (27.5, 31.9)	0.4[a]
Body fat (%), median (IQR)	23 $^{n=93}$ (19 to 29)	22 $^{n=83}$ (19 to 28)	0.6[a]
Muscle mass (kg), median (IQR)	73 $^{n=93}$ (68, 80)	78 $^{n=83}$ (72 to 82)	**0.012*[a]**
Smoking, never, *n* (%)	94 (93%)	85 (88%)	0.29[c]
Hypertension, >=140/90, *n* (%)	4 (4.0%)	5 (5.2%)	0.95[c]
Exercise, hours per week, median IQR	4.0 (2.5 to 6.5)	4.5 $^{n=94}$ (3.0 to 6.0)	0.5[a]
AUDIT raw score, median (IQR)	6 $^{n=97}$ (4 to 10)	7 $^{n=94}$ (5 to 12)	**0.048*[a]**
Alcohol consumption (AUDIT-C), median (IQR)	5 $^{n=97}$ (4 to 6)	5 $^{n=94}$ (4 to 7)	0.3[a]
Alcohol problematic consequences (AUDIT-P), median (IQR)	1 $^{n=97}$ (0 to 3)	2 $^{n=95}$ (0 to, 5)	**0.034*[a]**
Drug use (DUDIT), median (IQR)	0 $^{n=96}$ (0 ± 0)	0 $^{n=96}$ (0 ± 0)	**0.037*[a]**
Drug use ≥1 (DUDIT), *n* (%)	7/96 (6.9)	17/96 (17.5)	**0.05*[c]**
Player position, number of forwards (%)	55 (54.5%)	69 (71.1%)	**0.023*[c]**
Career duration, years, median (IQR)	10.0 (7.0 to 12.0)	10.0 (8.0 to 13.0)	0.55[a]
Microhaemorrhage, *n* (%)	3 (3.0)$^{n=100}$	3 (3.1)	1[c]
Cavum Septum Pellucidum grade 2 or above, *n* (%)	16 (15.8)$^{n=100}$	31 (32.0)	**0.014*[c]**

Table S1. Comparison of participant characteristics between low and high concussion groups (based on median split of 7 total career concussions). *n = 101 for low concussion group and n = 97 for high concussion group unless otherwise specified. IQR= interquartile range; OR = odds ratio; SD = standard deviation; r = Pearson correlation coefficient; Rho = Spearman's rank correlation coefficient; AUDIT = Alcohol Use Disorder Identification Test; DUDIT = Drug Use Disorder Identification Test.. [a]Wilcoxon signed-rank test. [b]Spearman's rank correlation. [c]Chi-squared test.. [d]T-test. *p-values <0.05.*

Symptom based questionnaires	Questionnaire scores			Proportion above cut-off, n/n (%)		
	Low concussion	High Concussion	p-value[a]	Low concussion	High Concussion	p-value[b]
**Self-reported questionnaire scores**						
Quality of life (EuroQol 5dim), median (IQR)	6.0 $^{n=96}$ (5.0 to 7.0)	7.0 $^{n=95}$ (5.0 to 9.0)	0.17			
Pain symptoms (EuroQol 5dim pain), median (IQR)	2.0 $^{n=96}$ (1.0 to 2.0)	2.0 $^{n=95}$ (1.0 to 2.0)	0.48			
Depression symptoms (BDI), median (IQR)	5.0 (2.0 to 11.0)	11.0 (5.5 to 18.0)	**0.001***	17/97 (17.5%)	37/95 (38.9%)	**0.005***
Anxiety symptoms (GAD7), median (IQR)	3.0 (1.0 to 5.0)	4.0 (2.0 to 9.0)	**0.003***	26/97 (26.8%)	41/95 (43.2%)	**0.039***
Total post-concussion symptoms (Modified Rivermead), median (IQR)	9.0 $^{n=96}$ (5.5 to 20.5)	19.0 $^{n=95}$ (10.0 to 28.0)	**0.001***			
Typical post-concussion symptoms (Modified Rivermead), median (IQR)	9.0 $^{n=95}$ (4.5 to 18.5)	18.0 $^{n=95}$ (10.0 to 24.5)	**0.001***			
Atypical post-concussion symptoms (Modified Rivermead), median (IQR)	0.0 $^{n=95}$ (0.0 to 1.0)	1.0 $^{n=95}$ (0.0 to 3.0)	**0.001***			
Sleep quality index score (PSQI), median (IQR)	6.0 (4.0 to 9.0)	6.0 (4.0 to 9.0)	0.48	53/97 (54.6%)	55/95 (57.9%)	0.76
Insomnia severity (ISI), median (IQR)	6.0 (2.0 to 9.0)	6.0 (2.0 to 10.0)	0.58	37/97 (38.1%)	38/94 (40.4%)	0.86
Self-reported behaviour rating of executive dysfunction (BRIEF-A GEC), median (IQR)	47.0 (41.0 to 57.0)	55.0 (46.0 to 64.0)	**0.001***	5/97 (5.2%)	21/93 (22.6%)	**0.0051***
**Informant-reported questionnaire scores**						
Behaviour rating of executive dysfunction (BRIEF-A GEC), median (IQR)	48.0 (42.0 to 54.0)	54.0 (44.0 to 60.0)	**0.003***	4/91 (4.4%)	14/85 (16.5&)	**0.034***
Neuropsychiatric symptom severity (NPIQ), median (IQR)	1.0 $^{n=86}$ (0.0 to 3.8)	3.0 $^{n=79}$ (0.5 to 7.0)	**0.015***			
Neuropsychiatric symptom caregiver distress (NPIQ), median (IQR)	1.0 $^{n=86}$ (0.0 to 4.0)	3.0 $^{n=79}$ (0.0 to 9.0)	0.058			

Table S2. Comparison of symptom-based questionnaires between low and high concussion groups (based on median split of 7 total career concussions). *n = 101 for low concussion group and n = 97 for high concussion group unless otherwise specified. Pre-existing clinically significant cut-offs were used for questionnaires where possible. Pre-existing cut-off scores: BDI ≥14; GAD7 ≥5; PSQI ≥6; ISI ≥8; BRIEF-A GEC ≥65. [a] Wilcoxon rank sum test.. [b]Chi-squared test . *p-values <0.05 (FDR corrected for multiple comparisons)*

Standardised neuropsychology	Test scores			Proportion impaired n/n (%)		
	Low concussion	High Concussion	p-value[a]	Low concussion	High Concussion	p-value[b]
Estimated premorbid functioning (FSIQ), mean (SD)	105.4 (7.7)	103.6 (8.0)	0.63			
Performance validity (Dot counting score), median (IQR)	9.3 (7.7 to 11.1)	9.7 (8.1 to 11.3)	0.91	2/101 (2%)	1/97 (1%)	0.80
Performance validity (Digit span, WAIS IV), median (IQR)	10.0 (9.0 to 12.0)	10.0 (9.0 to 11.0)	0.43	5/101 (5%)	3/97 (3.1%)	0.80
Executive function (Trail making test, B vs A ratio), median (IQR)	2.2 (1.8 to 2.5)	2.0 (1.8 to 2.6)	0.91			
Executive function (DKEFS inhibition switching vs baseline contrast), median (IQR)	30.2 (24.4 to 36.4)	31.1 (25.4 to 36.6)	0.91			
Auditory memory composite score (RBANS and WMS IV), mean (SD)	101.2 (11.4)	98.0 (11.7)	0.51	8/100 (8%)	8/97 (8.2%)	1
Processing speed index score (WAIS IV), mean (SD)	107.9 (12.9)	103.1 (11.6)	0.32	2/101 (2%)	6/97 (6.2%)	0.80
Working memory index score (WAIS IV), mean (SD)	113.3 (15.0)	109.5 (15.0)	0.43	0/101 (0%)	1/97 (1%)	1
Attention index score (RBANS), mean (SD)	108.3 (16.3)	101.8 (13.7)	0.12	3/101 (3%)	6/97 (6.2%)	0.80
Language index score (RBANS), mean (SD)	104.8 (11.7)	104.6 (10.1)	0.91	2/101 (2%)	1/97 (1%)	0.88
Visuospatial index score (RBANS), mean (SD)	115.8 (11.0)	114.9 (10.1)	0.91	1/101 (1%)	1/97 (1%)	1

Table S3. Comparison of standardised neuropsychology tests between low and high concussion groups (based on median split of 7 total career concussions). *n = 101 for low concussion group and n = 97 for high concussion group unless otherwise specified. FSIQ = Full-Scale Intelligence Quotient; WAIS IV = Wechsler Adult Intelligence Scale, Fourth edition; DKEFS = Delis-Kaplan Executive Function System; RBANS = Repeatable Battery for the Assessment of Neuropsychological Status; WMS IV = Wechsler Memory Scale – Fourth edition. Cognitive impairment on neuropsychology tasks were defined by an index score that is ≥1.5 standard deviations below each individual's FSIQ. [a]Linear regression adjusted for age, education years and sex. [b]Logistic regression adjusted for age, education years and sex. *p-values <0.05 FDR corrected*

Cognitron computerised neuropsychology	Raw scores			Deviation from Expected score		
	Low concussion	High concussion	p-value	Low concussion	High concussion	p-value
Objects immediate memory accuracy, mean (SD)	47.9 (6.7)	48.7 (6.0)	0.66[a]	-0.17 (0.91)	-0.28 (1.01)	0.68[c]
Objects immediate memory duration (seconds), mean (SD)	159.7 (26.2)	160.7 (27.5)	0.72[b]	-0.44 (0.17)	-0.45 (0.17)	0.81[c]
Objects delayed memory accuracy, mean (SD)	46.5 (7.0)	47.9 (6.0)	0.36[a]	-0.06 (1.02)	-0.18 (0.97)	0.68[c]
Objects delayed memory duration (seconds), mean (SD)	81.1 (13.4)	83.4 (15.4)	0.56[b]	-0.08 (0.32)	-0.12 (0.26)	0.80[c]
Tower of London accuracy, mean (SD)	6.8 (2.1)	7.3 (1.8)	0.36[a]	0.09 (0.78)	-0.10 (0.82)	0.40[c]
Tower of London RT (seconds), mean (SD)	12.5 (4.1)	13.4 (5.5)	0.36[b]	0.41 (1.05)	0.25 (0.80)	0.43[c]
2D Manipulations accuracy, mean (SD)	28.9 (7.2)	30.6 (8.3)	0.36[a]	0.44 (1.04)	0.20 (0.89)	0.40[c]
2D Manipulations RT (seconds), mean (SD)	4.6 (1.5)	4.4 (1.6)	0.36[b]	-0.24 (0.86)	-0.09 (0.83)	0.43[c]
Simple reaction time (milliseconds), mean (SD)	321.9 (48.7)	316.2 (42.4)	0.19[b]	-0.22 (0.73)	-0.08 (0.87)	043[c]
Choice reaction time (milliseconds), mean (SD)	488.0 (50.8)	478.2 (44.0)	0.36[b]	0.03 (0.52)	0.20 (0.60)	0.40[c]

Table S4. Comparison of computerized neuropsychology tests between low and high concussion groups (based on median split of 7 total career concussions). *n = 101 for low concussion group and n = 97 for high concussion group. [a]Linear regression adjusted for age, education years and sex. [b]generalised linear model with gamma log link function. [c]Deviation from Expected' (DfE) scores derived from linear models trained to the normative dataset to predict each score based on a range of demographic factors (i.e., age, age squared, gender, education, language, handedness, and device)[39,47] group comparisons performed using general linear models.*

	Questionnaire scores	
**Symptom based questionnaires**	**Rho**	**p-value[a]**
**Self-reported questionnaire scores**		
Quality of life (EuroQol 5dim), median (IQR)	-0.016	0.99
Pain symptoms (EuroQol 5dim pain), median (IQR)	-0.021	0.99
Depression symptoms (BDI), median (IQR)	-0.012	0.99
Anxiety symptoms (GAD7), median (IQR)	-0.050	0.99
Total post-concussion symptoms (Modified Rivermead), median (IQR)	0.001	0.99
Typical post-concussion symptoms (Modified Rivermead), median (IQR)	-0.014	0.99
Atypical post-concussion symptoms (Modified Rivermead), median (IQR)	0.129	0.97
Sleep quality index score (PSQI), median (IQR)	-0.027	0.99
Insomnia severity (ISI), median (IQR)	0.003	0.99
Self-reported behaviour rating of executive dysfunction (BRIEF-A GEC), median (IQR)	-0.034	0.99
**Informant-reported questionnaire scores**		
Behaviour rating of executive dysfunction (BRIEF-A GEC), median (IQR)	0.044	0.99
Neuropsychiatric symptom severity (NPIQ), median (IQR)	-0.079	0.99
Neuropsychiatric symptom caregiver distress (NPIQ), median (IQR)	0.032	0.99

Table S5. Relationship between symptom-based questionnaires and career duration. *Pre-existing clinically significant cut-offs were used for questionnaires where possible.. [a]Spearman's correlation.* ****p-values <0.05*** *(FDR corrected for multiple comparisons)*

Standardised neuropsychology	Test scores		Impaired	
	Regression coefficient	p-value[a]	Odd's ratio	p-value[b]
Estimated premorbid functioning (FSIQ)	-0.32	0.17		
Performance validity (Dot counting score)	0.01	0.84	0.021	0.9
Performance validity (Digit span, WAIS IV)	-0.04	0.44	-0.075	0.9
Executive function (Trail making test, B vs A ratio),	0.00	0.84		
Executive function (DKEFS inhibition switching vs baseline contrast)	-0.06	0.84		
Auditory memory composite score (RBANS and WMS IV)	-0.26	0.4	0.041	0.9
Processing speed index score (WAIS IV)	-0.48	0.17	-0.069	0.9
Working memory index score (WAIS IV)	-0.37	0.4	0.362	0.9
Attention index score (RBANS)	-0.38	0.4	-0.014	0.9
Language index score (RBANS)	-0.29	0.4	0.095	0.9
Visuospatial index score (RBANS)	-0.18	0.46	-0.093	0.9

Table S6. Relationship between career duration and performance on standardised neuropsychology tests. FSIQ *= Full-Scale Intelligence Quotient; WAIS IV = Wechsler Adult Intelligence Scale, Fourth edition; DKEFS = Delis-Kaplan Executive Function System; RBANS = Repeatable Battery for the Assessment of Neuropsychological Status; WMS IV = Wechsler Memory Scale – Fourth edition. Cognitive impairment on neuropsychology tasks were defined by an index score that is ≥1.5 standard deviations below each individual's FSIQ. [a]Linear regression adjusted for age, education years and sex. [b]Logistic regression adjusted for age, education years and sex. *p-values <0.05 FDR corrected.*

Cognitron computerised neuropsychology	Raw scores		Deviation from Expected score	
	Co-efficient	p-value	Co-efficient	p-value
Objects immediate memory accuracy	0.002	0.98[a]	-0.03	0.97[c]
Objects immediate memory duration (seconds)	-0.0003	0.91[b]	151,4	0.97[c]
Objects delayed memory accuracy	0.03	0.78[a]	-0.02	0.97[c]
Objects delayed memory duration (seconds)	0.10	0.58[b]	274.1	0.82[c]
Tower of London accuracy	-0.004	0.90[a]	-0.001	0.97[c]
Tower of London RT (seconds	-0.01	0.23[b]	-72.02	0.82[c]
2D Manipulations accuracy	-0.01	0.95[a]	-0.13	0.82[c]
2D Manipulations RT (seconds)	0.001	0.83[b]	29.89	0.82[c]
Simple reaction time (milliseconds)	0.001	0.70[b]	0.54	0.82[c]
Choice reaction time (milliseconds)	-0.001	0.69[b]	0.66	0.82[c]

Table S7. Relationship between career duration and performance on computerized neuropsychology *[a]Linear regression adjusted for age, education years and sex. [b]generalised linear model with gamma log link function adjusted for age, education years and sex. [c]Deviation from Expected' (DfE) scores derived from linear models trained to the normative dataset to predict each score based on a range of demographic factors (i.e., age, age squared, gender, education, language, handedness, and device)[39,47] relationship with career duration performed using general linear model. *p-values <0.05 (FDR corrected).*

	Backs (n=74)	Forwards (n=126)	p-value
Age, years, median (IQR)	45.0 (39.0 to 50.0)	44.0 (38.2 to 51.0)	0.73[a]
Male sex, *n* (%)	65 (87.8)	116 (92.1)	0.46[c]
Years of education, median (IQR)	16.0 (16.0 to 17.0)	16.0 (13.2 to 17.0)	0.067[a]
Ethnicity, *n* (%)			**0.03**[*c]
White	67 (90.5)	123 (97.6)	
Black Caribbean	1 (1.4)	0 (0.0)	
Black African	3 (4.1)	0 (0.0)	
Other ethnic group (including 'mixed')	3 (4.1)	1 (0.8)	
Not stated	0 (0.0)	2 (1.6)	
Weight, kg, median (IQR)	92.8 (85.7 to 97.6)	109 (100 to 123)	**<0.001**[*a]
Height, cm, mean (SD)	180.4 (7.1)	188 (8.9)	**<0.001**[*e]
BMI, median (IQR)	28.1 (26.2 to 29.5)	30.1 (27.8 to 34.5)	**<0.001**[*a]
Body fat (%), median (IQR)	20 $^{n=67}$ (18 to 23)	26 $^{n=110}$ (21 to 31)	**<0.001**[*a]
Muscle mass (kg), median (IQR)	69 $^{n=67}$ (66 to 72)	79 $^{n=110}$ (75 to 83)	**<0.001**[*a]
Smoking, never, *n* (%)	69 (93%)	112 (89%)	0.44[c]
Hypertension, >=140/90, *n* (%)	2 (2.7%)	8 (6.3%)	0.42[c]
Exercise, hours per week, median IQR	5.0 (3.0 to 6.9)	4.0 $^{n=123}$ (3.0 to 6.0)	0.3[a]
AUDIT raw score, median (IQR)	6 $^{n=71}$ (4 to 11)	7 $^{n=121}$ (5 to 11)	0.5[a]
Alcohol consumption (AUDIT-C), median (IQR)	5 $^{n=71}$ (4 to 5) $^{n=71}$	5 $^{n=121}$ (4 to 7)	0.4[a]
Alcohol problematic consequences (AUDIT-P), median (IQR)	1 $^{n=72}$ (0 to 3)	2 $^{n=121}$ (0 to 4)	0.5[a]
Drug use (DUDIT), median (IQR)	0 $^{n=72}$ (0 ± 0)	0 $^{n=121}$ (0 ± 0)	0.2[a]
Drug use ≥1 (DUDIT), *n* (%)	6/72 (8.1)	18/121 (14.3)	0.3[c]
Self-reported concussions, median (IQR)	6.0 (3.0 to 11.8)	9.0 $^{n=124}$ (4.0 to 20.0)	**0.011**[*a]
Career duration, years, median (IQR)	10.0 (7.0 to 12.0)	11.0 (8.0 to 13.0)	0.23[a]
Microhaemorrhage, *n* (%)	4 (5.4) $^{n=73}$	2 (1.6)	0.26[c]
Cavum Septum Pellucidum grade 2 or above, *n* (%)	17 (23.0) $^{n=73}$	31 (24.6)	0.97[c]

Table S8. Comparison of participant characteristics between backs and forwards. *n = 74 backs and n = 126 forwards unless otherwise specified IQR= interquartile range;* r_s *= Spearman's rank correlation coefficient; OR = odds ratio; SD = standard deviation; r = Pearson correlation coefficient; AUDIT = Alcohol Use Disorder Identification Test; DUDIT = Drug Use Disorder Identification Test; LOC = loss of consciousness.* [a]*Wilcoxon signed-rank test.* [b]*Spearman's rank correlation.* [c]*Chi-squared test.* [d]*Logistic regression.* [e]*T-test.* [f]*Pearson correlation. *p-values <0.05.*

	Questionnaire scores			Proportion above cut-off, n/n (%)		
**Symptom based questionnaires**	**Backs**	**Forwards**	**p-value[a]**	**Backs**	**Forwards**	**p-value[b]**
**Self-reported questionnaire scores**						
Quality of life (EuroQol 5dim), median (IQR)	6.0 $^{n=72}$ (5.0 to 8.0)	7.0 $^{n=120}$ (5.8 to 8.2)	0.44			
Pain symptoms (EuroQol 5dim pain), median (IQR)	2.0 $^{n=72}$ (1.0 to 2.0)	2.0 $^{n=120}$ (1.0 to 2.0)	1			
Depression symptoms (BDI), median (IQR)	7.0 (2.0 to 14.0)	9.0 $^{n=120}$ (4.0 to 15.2)	0.23	19/73 (26%)	36/120 (30%)	0.8
Anxiety symptoms (GAD7), median (IQR)	3.0 (1.0 to 6.0)	3.0 (2.0 to 6.0)	0.77	29/73 (39.7%)	39/120 (32.5%)	0.66
Total post-concussion symptoms (Modified Rivermead), median (IQR)	8.0 $^{n=72}$ (4.0 to 24.2)	16.0 $^{n=119}$ (9.0 to 25.5)	**0.013***			
Typical post-concussion symptoms (Modified Rivermead), median (IQR)	8.0 $^{n=72}$ (4.0 to 21.2)	15.0 $^{n=119}$ (9.0 to 22.0)	**0.013***			
Atypical post-concussion symptoms (Modified Rivermead), median (IQR)	0.0 $^{n=72}$ (0.0 to 1.0)	0.0 $^{n=119}$ (0.0 to 2.5)	0.21			
Sleep quality index score (PSQI), median (IQR)	6.0 (4.0 to 9.0)	6.0 (4.0 to 9.0)	0.60	39/73 (53.4%)	70/120 (58.3%)	0.61
Insomnia severity (ISI), median (IQR)	5.0 (2.0 to 12.0)	6.0 (2.5 to 9.0)	0.77	28/73 (38.4%)	48/119 (40.3%)	0.9
Self-reported behaviour rating of executive dysfunction (BRIEF-A GEC), median (IQR)	48.0 (39.0 to 57.0)	53.0 (44.0 to 62.0)	**0.04***	7/71 (9.9%)	20/120 (16.7%)	0.66
**Informant-reported questionnaire scores**						
Behaviour rating of executive dysfunction (BRIEF-A GEC), median (IQR)	48.0 (42.2 to 54.8)	53.0 (44.0 to 58.0)	0.11	5/66 (7.6%)	14/112 (12.5%)	0.66
Neuropsychiatric symptom severity (NPIQ), median (IQR)	1.0 $^{n=60}$ (0.0 to 4.0)	3.0 $^{n=107}$ (0.5 to 6.0)	0.21			
Neuropsychiatric symptom caregiver distress (NPIQ), median (IQR)	1.0 $^{n=60}$ (0.0 to 4.0)	2.0 $^{n=107}$ (0.0 to 7.5)	0.23			

Table S9. Comparison of symptom-based questionnaires between backs and forwards. *n = 74 backs and n = 126 forwards unless otherwise specified in the 'Proportion above cut-off column' total. Pre-existing clinically significant cut-offs were used for questionnaires where possible. Pre-existing cut-off scores: BDI ≥14; GAD7 ≥5; PSQI ≥6; ISI ≥8; BRIEF-A GEC ≥65. [a] Wilcoxon rank sum test.. [b]Chi-squared test . *p-values <0.05 (FDR corrected for multiple comparisons)*

	Test scores			Proportion impaired n/n (%)		
**Standardised neuropsychology**	**Backs**	**Forwards**	**p-value[a]**	**Backs**	**Forwards**	**p-value[b]**
Estimated premorbid functioning (FSIQ), mean (SD)	105.9 (7.3)	103.7 (8.2)$^{n=125}$	0.19			
Performance validity (Dot counting score), median (IQR)	8.7 (7.4 to 11.7)	9.7 (8.3 to 11.2)	0.19	1/74 (1.4%)	2/125 (1.6%)	1
Performance validity (Digit span, WAIS IV), median (IQR)	10.0 (9.0 to 12.0)	10.0 (9.0 to 11.0)	0.28	2/74 (2.7%)	6/125 (4.8%)	1
Executive function (Trail making test, B vs A ratio), median (IQR)	2.1 (1.8 to 2.4)	2.2 $^{n=124}$ (1.8 to 2.6)	0.35			
Executive function (DKEFS inhibition switching vs baseline contrast), median (IQR)	30.4 (23.5 to 36.1)	31.1 $^{n=124}$ (26.0 to 36.7)	0.34			
Auditory memory composite score (RBANS and WMS IV), mean (SD)	101.7 (12.0)	98.8 (16.0)	0.28	5/73 (6.8%)	11/125 (8.8%)	1
Processing speed index score (WAIS IV), mean (SD)	109.0 (13.6)	103.3 (11.4)	0.065	1/74 (1.4%)	7/125 (5.6%)	1
Working memory index score (WAIS IV), mean (SD)	114.8 (14.4)	109.3 (15.2)	0.13	0/74 (0%)	1/125 (0.8%)	1
Attention index score (RBANS), mean (SD)	107.7 (14.8)	103.5 (15.5)	0.19	2/74 (2.7%)	7/125 (5.6%)	1
Language index score (RBANS), mean (SD)	105.7 (11.0)	104.1 (10.8)	0.5	0/74 (0%)	3/125 (2.4%)	1
Visuospatial index score (RBANS), mean (SD)	117.0 (10.0)	114.4 (10.8)	0.19	0/74 (0%)	2/125 (1.6%)	1

Table S10. Comparison of standardised neuropsychology tests between backs and forwards. *n = 74 backs and n = 126 forwards unless otherwise specified in the 'Proportion above cut-off column' total. FSIQ = Full-Scale Intelligence Quotient; WAIS IV = Wechsler Adult Intelligence Scale, Fourth edition; DKEFS = Delis-Kaplan Executive Function System; RBANS = Repeatable Battery for the Assessment of Neuropsychological Status; WMS IV = Wechsler Memory Scale – Fourth edition. Cognitive impairment on neuropsychology tasks were defined by an index score that is ≥1.5 standard deviations below each individual's FSIQ. [a]Linear regression adjusted for age, education years and sex. [b]Logistic regression adjusted for age, education years and sex. *p-values <0.05 FDR corrected.*

Cognitron computerised neuropsychology	Raw scores			Deviation from Expected score		
	Backs	Forwards	p-value	Backs	Forwards	p-value
Objects immediate memory accuracy, mean (SD)	48.5 (6.7)	48.0 (6.5)	0.92[a]	-0.21 (0.97)	-0.26 (0.98)	0.94[c]
Objects immediate memory duration (seconds), mean (SD)	160.2 (26.7)	160.1 (26.4)	0.92[b]	-0.44 (0.16)	-0.44 (0.16)	0.94[c]
Objects delayed memory accuracy, mean (SD)	47.5 (5.4)	46.9 (7.1)	0.92[a]	-0.16 (-0.16)	-0.13(0.98)	0.94[c]
Objects delayed memory duration (seconds), mean (SD)	81.6 (13.5)	82.2 (14.4)	0.92[b]	-0.13 (0.32)	-0.10 (0.28)	0.83[c]
Tower of London accuracy, mean (SD)	7.4 (1.8)	7.0 (2.1)	0.63[a]	0.07 (0.83)	-0.03 (0.83)	0.82[c]
Tower of London RT (seconds), mean (SD)	13.0 (4.6)	12.9 (4.8)	0.92[b]	0.33 (0.90)	0.33 (0.92)	0.94[c]
2D Manipulations accuracy, mean (SD)	30.6 (8.0)	29.3 (7.9)	0.70[a]	30.6 (8.0)	0.27 (0.99)	0.75[c]
2D Manipulations RT (seconds), mean (SD)	4.3 (1.4)	4.7 (1.6)	0.63[b]	4.3 (1.4)	-0.10(0.88)	0.67[c]
Simple reaction time (milliseconds), mean (SD)	306.7 (38.4)	324.9 (45.7)	**0.05***[b]	-0.37 (0.69)	-0.05 (0.81)	**0.05***[c]
Choice reaction time (milliseconds), mean (SD)	467.3 (39.5)	491.6 (47.4)	**0.01***[b]	-0.07 (0.50)	0.21 (0.58)	**0.03***[c]

Table S11. Comparison of computerized neuropsychology tests between retired backs and forwards. *n = 74 backs and n= 126 forwards. [a]Linear regression adjusted for age, education years and sex. [b]generalised linear model with gamma log link function *p-values <0.05 (FDR corrected)*
